# Comparatively Barcoded Chromosomes of *Brachypodium* Perennials Tell the Story of Their Karyotype Structure and Evolution

**DOI:** 10.3390/ijms20225557

**Published:** 2019-11-07

**Authors:** Joanna Lusinska, Alexander Betekhtin, Diana Lopez-Alvarez, Pilar Catalan, Glyn Jenkins, Elzbieta Wolny, Robert Hasterok

**Affiliations:** 1 Institute of Biology, Biotechnology and Environmental Protection, Faculty of Natural Sciences, University of Silesia in Katowice, 40-032 Katowice, Poland; jlusinska@us.edu.pl (J.L.); alexander.betekhtin@us.edu.pl (A.B.); 2 Faculty of Agricultural Sciences, National University of Columbia, Palmira 763533, Colombia; dianalopez430@gmail.com; 3 Department of Agriculture (Botany), High Polytechnic School of Huesca, University of Zaragoza, 22071 Huesca, Spain; pilar.catalan09@gmail.com; 4 Grupo de Bioquímica, Biofísica y Biología Computacional (BIFI, UNIZAR), Unidad Asociada al CSIC, 50018 Zaragoza, Spain; 5 Institute of Biology, Tomsk State University, Tomsk 634050, Russia; 6 Institute of Biological, Environmental and Rural Sciences, Aberystwyth University, Aberystwyth SY23 3DA, UK; gmj@aber.ac.uk

**Keywords:** *Brachypodium*, comparative chromosome barcoding, dysploidy, karyotype structure and evolution, model grass genus, molecular cytogenetics, polyploidy

## Abstract

The *Brachypodium* genus is an informative model system for studying grass karyotype organization. Previous studies of a limited number of species and reference chromosomes have not provided a comprehensive picture of the enigmatic phylogenetic relationships in the genus. Comparative chromosome barcoding, which enables the reconstruction of the evolutionary history of individual chromosomes and their segments, allowed us to infer the relationships between putative ancestral karyotypes of extinct species and extant karyotypes of current species. We used over 80 chromosome-specific BAC (bacterial artificial chromosome) clones derived from five reference chromosomes of *B. distachyon* as probes against the karyotypes of twelve accessions representing five diploid and polyploid *Brachypodium* perennials. The results showed that descending dysploidy is common in *Brachypodium* and occurs primarily via nested chromosome fusions. *Brachypodium* *distachyon* was rejected as a putative ancestor for allotetraploid perennials and *B. stacei* for *B. mexicanum.* We propose two alternative models of perennial polyploid evolution involving either the incorporation of a putative *x* = 5 ancestral karyotype with different descending dysploidy patterns compared to *B. distachyon* chromosomes or hybridization of two *x* = 9 ancestors followed by genome doubling and descending dysploidy. Details of the karyotype structure and evolution in several *Brachypodium* perennials are revealed for the first time.

## 1. Introduction

In recent years, the genus *Brachypodium* has become one of the most comprehensively studied genera among monocotyledonous plants primarily due to the exploitation of one of its flagship species, *B. distachyon*, as a functional model organism for temperate cereals and other economically important grasses [1,2,3,4,5]. It comprises three annuals, the diploids *B. distachyon* (2*n* = 10) and *B. stacei* (2*n* = 20) and their derived allotetraploid *B. hybridum* (2*n* = 30), which have recently been proposed as a model system to study polyploidy and grass speciation [6,7]. Approximately 15 other representatives are perennials distributed worldwide [5,8]. All *Brachypodium* species have small and compact nuclear genomes, diverse (*x* = 5, 8, 9, 10) basic chromosome numbers and various ploidy levels [9,10,11,12], which are accompanied by complicated and still not fully resolved phylogenetic relationships [8,13,14]. Recent phylogenetic studies that were based mainly on combined analyses of some nuclear and plastid genes dated the origin and split of the crown *Brachypodium* ancestor in the Mid-Miocene (12.6 Ma) [6,8,13,15]. *Brachypodium* perennials are very diverse in terms of their phenotype, origin, and geographical distribution. They range from the American short-rhizomatous *B. mexicanum* (2*n* = 40), which resembles the annual more than the perennial taxa [8,16], to the more recently evolved Eurasian and African long-rhizomatous diploid and allopolyploid species of the core-perennial clade, i.e., *B. arbuscula* (2*n* = 18), *B. boissieri* (2*n* = 42, 46), *B. glaucovirens* (2*n* = 16), *B. phoenicoides* (2*n* = 28), *B. pinnatum* (2*n* = 16, 18, 28)*, B. retusum* (2n = 36, 38), *B. rupestre* (2*n* = 18, 28), and *B. sylvaticum* (2*n* = 18) [3,6,8,10,17]. One of the most widespread and best studied perennial species is *B. sylvaticum* which has considerable readily available genetic resources such as inbred lines, efficient transformation protocols, and genomic and transcriptomic tools. Because of these, it has been recently proposed as a new model plant to study perenniality [3,7,18,19]. In some earlier reports, *B. sylvaticum* was used to assist the molecular characterization of the *Ph1* locus in wheat [20] and to compare gene conservation and collinearity with orthologous regions from rice and wheat [21]. Given the economic importance of perennial grasses, comparative studies of more closely related *Brachypodium* annuals and perennials can also be of particular importance in identifying and testing candidate “perenniality” genes and creating a tractable model system for both fundamental research and crop improvement [7,18].

Synteny-based paleogenomics implies that the present-day karyotypes originated from ancestral genomes with the lowest number of historical polyploidization events [22,23]. This offers insight into the putative numbers of protochromosomes of the respective progenitors of the current species and provides an opportunity to link the karyotypes of extant species, including that of *B. distachyon,* with those of their hypothetical and extinct ancestors [4,24,25]. Comparative genomics identifies polyploidization and dysploidy events, which are complemented by minor genome rearrangements, as crucial factors in the evolution, divergence, and adaptive speciation of all flowering plants [26,27]. Despite its great importance, our ability to understand polyploid genome evolution, including that of economically important crops, is still constrained by a limited knowledge of the actual parents and incomplete lineage reconstruction during polyploid speciation [7,28]. Within the complexity of many plant species, polyploid series are often described either as intraspecific cytotypes showing different ploidy levels and very similar morphological features [29,30] or are considered different species [31]. For example, *B. distachyon* was initially described as a species with three cytotypes comprising an *x* = 5 basic chromosome number [11]. However, the seminal cytogenetic analyses of Hasterok et al. [32,33], coupled with later molecular studies [34] and a comprehensive taxonomic description and phylogenetic analysis [6], drove their reclassification into three separate species (*B. distachyon, B. stacei*, and *B. hybridum*). Moreover, fluorescence in situ hybridization (FISH)-based studies, including comparative chromosome painting (CCP) and comparative chromosome barcoding (CCB), concluded that various *Brachypodium* allopolyploids were derived from interspecific crosses of distinct diploid, perennial, and/or annual progenitors [9,12,35]. Although the exact taxonomic identity of the diploid (2*n* = 16, 18) and allotetraploid (2*n* = 28) cytotypes of *B. pinnatum* and *B. rupestre* remains unclear, there is a growing body of evidence [9,12,13] that they should be classified as separate species, thereby paralleling the case of the diploid–allotetraploid *B. distachyon* complex [6,32].

The availability of the *B. distachyon* whole-genome sequence [4] (https://phytozome.jgi.doe.gov/pz/portal.html#!info?alias=Org_Bdistachyon) combined with FISH using low-repeat BAC (bacterial artificial chromosomes) clones as probes [36,37,38] enabled the precise dissection of the chromosome structure at the microscopic level via selective visualization of either their smaller (via CCB) or larger (including entire chromosomes: via CCP) regions. Apart from *Brachypodium*, similar approaches are limited in plants to a handful of small-genome taxa within Brassicaceae [39,40], *Cucumis* [41], rice [42], and, recently, also in maize [43]. To date, detailed analyses of the karyotype structure and evolution using CCB in *Brachypodium* essentially targeted the annuals [44]. The relatively few studies on perennials have been constrained in the past by a paucity of chromosome markers and unavailability of germplasm [14,35,45]. We address these technical obstacles here and present a comprehensive model of the karyotype structure and evolution of perennial *Brachypodium* species.

## 2. Results

**Figure 1:**
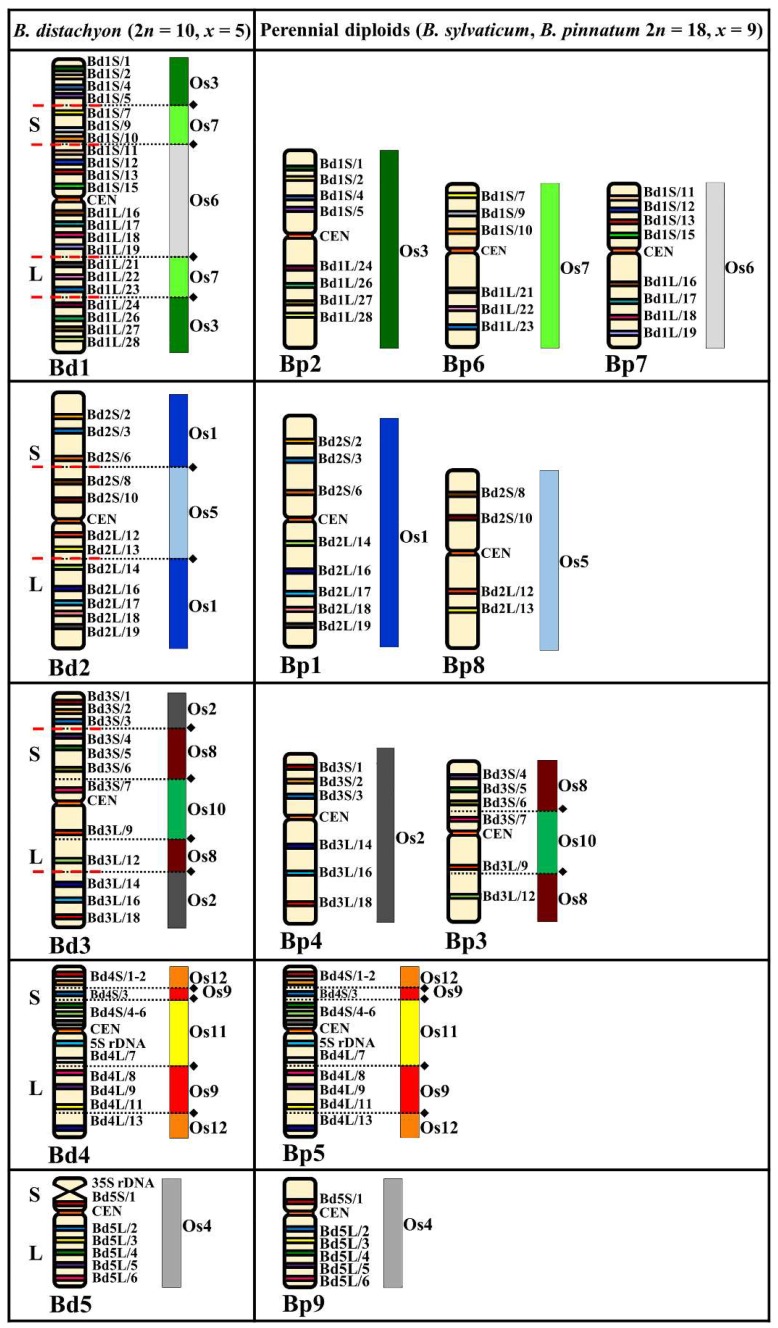
Distribution of the bacterial artificial chromosome (BAC) clones derived from chromosomes Bd1–Bd5 of *B. distachyon* (2*n* = 10, *x* = 5) that were comparatively mapped to the chromosomes of the *Brachypodium* perennial diploids (*B. sylvaticum* and *B. pinnatum*, 2*n* = 18, *x* = 9). Only one homologue from a pair is shown. The diagrams next to the *Brachypodium* (Bd, Bp) chromosomes align the BAC clones to the homoeologous regions (syntenic segments) in the relevant ancestral rice chromosome equivalents (ARCEs), Os1–Os12. Black diamonds and dotted lines indicate the hypothetical fusion points of the ARCE (adapted from IBI, [4]). Red, dashed lines indicate the chromosomal breakpoints in the Bp-genome chromosomes in *B. sylvaticum* and *B. pinnatum* 2*n* = 18 that were found by comparative chromosome barcoding.

**Figure 2:**
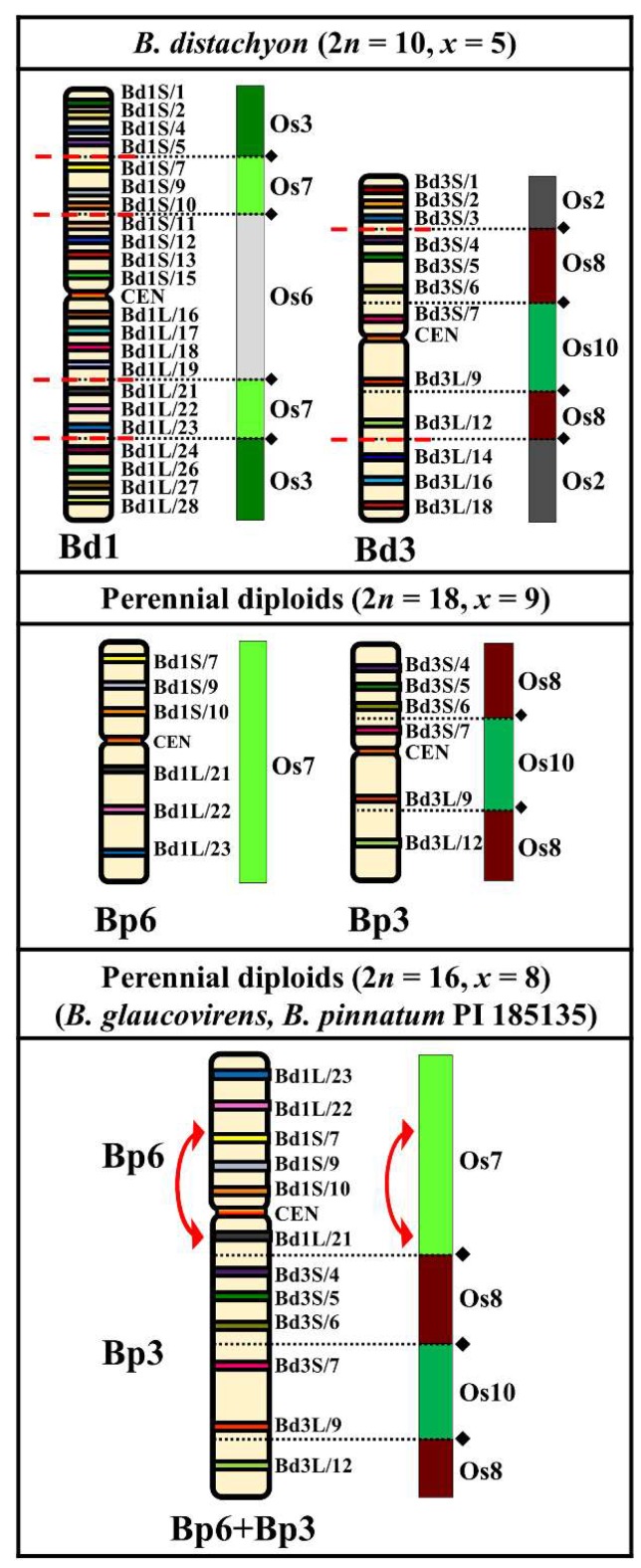
Distribution of the bacterial artificial chromosome (BAC) clones derived from chromosomes Bd1 and Bd3 of *B. distachyon* (2*n* = 10, *x* = 5) that were comparatively mapped to chromosomes Bp6 and Bp3 of the *Brachypodium* perennial diploids (2*n* = 18, *x* = 9) and to the chromosome Bp6+Bp3 of *B. glaucovirens* and *B. pinnatum* PI 185135 (both 2*n* = 16, *x* = 8). Only one homologue from a pair is shown. The diagrams next to the *Brachypodium* (Bd, Bp) chromosomes align the BAC clones to the homoeologous regions (syntenic segments) in the relevant ancestral rice chromosome equivalents (ARCEs). Black diamonds and dotted lines indicate the hypothetical fusion points of the ARCE (adapted from IBI, [4]). Red, dashed lines indicate the chromosomal breakpoints in the Bp-genome chromosomes of *B. sylvaticum* and *B. pinnatum* (2*n* = 18) that were found by comparative chromosome barcoding. The diagram for the *x* = 8 perennial diploids shows the specific end-to-end translocation of the putative Bp6 and Bp3 chromosomes which led to the formation of a specific Bp6+Bp3 chromosome. Red arrow points to the pericentric inversion that was found on this chromosome in *B. glaucovirens* and *B. pinnatum* PI 185135 2*n* = 16.

**Figure 3:**
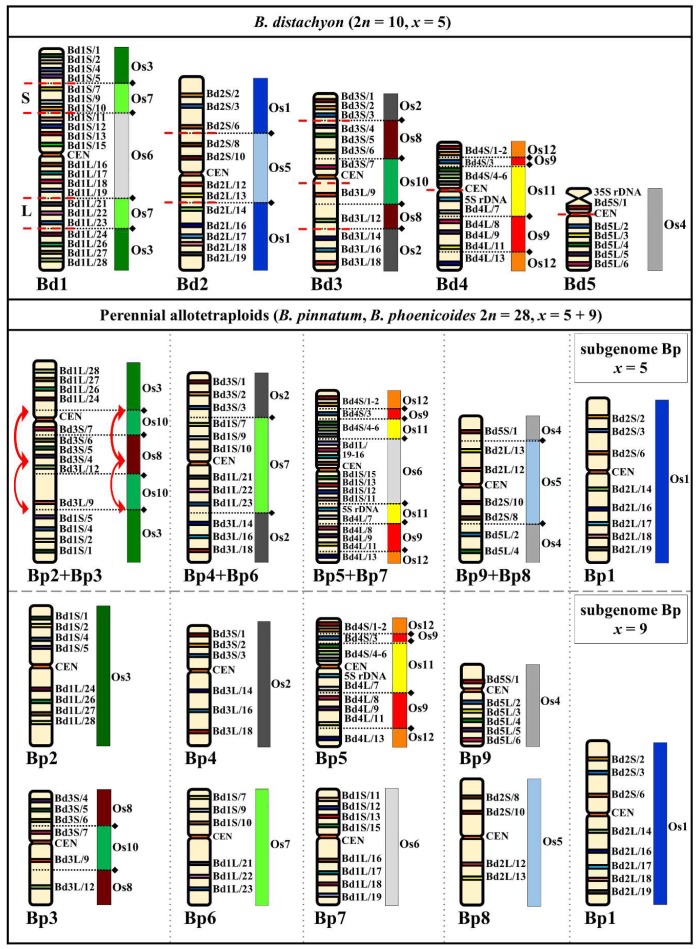
Distribution of the bacterial artificial chromosome (BAC) clones derived from chromosomes Bd1–Bd5 of *B. distachyon* (2*n* = 10, *x* = 5) that were comparatively mapped to the chromosomes of the *Brachypodium* perennial allotetraploids (2*n* = 28, *x* =5 + 9). Only one homologue from a pair is shown. The diagrams next to the *Brachypodium* (Bd, Bp) chromosomes align the BAC clones to the homoeologous regions (syntenic segments) in the relevant ancestral rice chromosome equivalents (ARCEs), Os1–Os12. Black diamonds and dotted lines indicate the hypothetical fusion points of the ARCE (adapted from IBI, [4]). Red, dashed lines indicate the chromosomal breakpoints in the chromosomes of two Bp subgenomes in *B. pinnatum* 2*n* = 28 and *B. phoenicoides* that were found by comparative chromosome barcoding. Red arrows point to the most likely one pericentric and one paracentric inversion that were found in chromosome Bp2+Bp3 in *B. pinnatum* 2*n* = 28 and *B. phoenicoides*.

**Figure 4:**
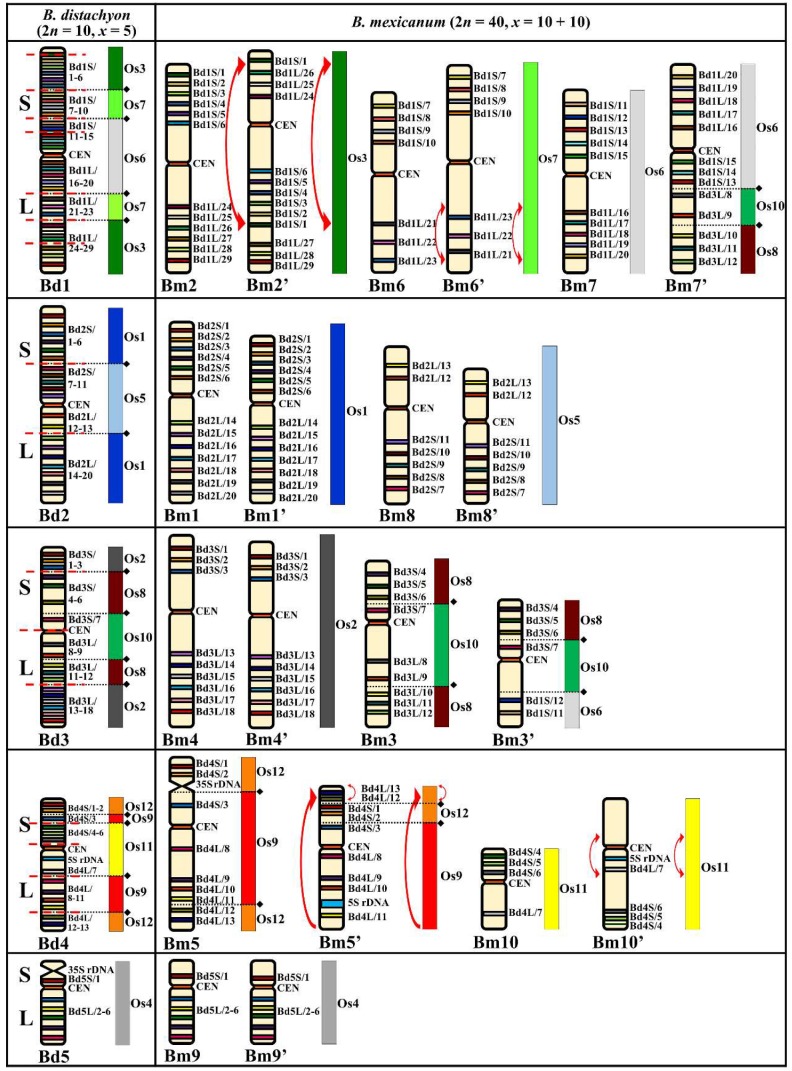
Distribution of the bacterial artificial chromosome (BAC) clones derived from chromosomes Bd1–Bd5 of *B. distachyon* (2*n* = 10, *x* = 5) that were comparatively mapped to the chromosomes of *B. mexicanum* (2*n* = 40, *x* = 10 + 10). Only one homologue from a pair is shown. The diagrams next to the *Brachypodium* (Bd, Bm) chromosomes align the BAC clones to the homoeologous regions (syntenic segments) in the relevant ancestral rice chromosome equivalents (ARCEs), Os1–Os12. Black diamonds and dotted lines indicate the hypothetical fusion points of the ARCEs (adapted from IBI, [4]). Red, dashed lines indicate the chromosomal breakpoints in the Bm-subgenome and Bm’-subgenome chromosomes of *B. mexicanum* that were found by comparative chromosome barcoding. Red arrows point to a pericentric inversion combined with a duplication of the region hybridizing with clone Bd1S/1 that was found on chromosome Bm2′; the paracentric inversion in the long arm of Bm6′; the translocation connected with the inversion of the terminal fragment containing clones Bd4/L12–13 in Bm5′; and a small pericentric inversion in the proximal region of chromosome Bm10′ that involves 5S rDNA and Bd4L/7 loci.

The karyotypes of both diploid and allopolyploid *Brachypodium* perennials (Table 1) were compared using the CCB mapping approach and with reference to *B. distachyon*. The use of 86 low-repeat BAC clones as the FISH probes for *B. mexicanum* and 59 clones for other *Brachypodium* perennial species enabled us to precisely track and analyze the evolutionary rearrangements of individual chromosomes and, consequently, entire karyotypes. We used differentially labelled, overlapping triplets of single-locus BACs at contiguous positions on the physical map of a particular chromosome of *B. distachyon* (Table S1). To clarify the relationships among the mapped chromosomal regions of the *Brachypodium* karyotypes, additional FISH experiments were performed with specific non-adjacent pairs of single-locus BAC-based probes and a centromeric BAC BD_CBa0033J12 (CEN). The results of the CCB were analyzed with reference to the so-called Bd-genome of *B. distachyon* and published genomic data from the whole-genome comparison of *B. distachyon* and rice [4]. Cytogenetic maps of the chromosomes were constructed based upon the results of the cross-species chromosome mapping (Figure 1, Figure 2, Figure 3 and Figure 4). We adopted the nomenclature for the chromosomes of the *Brachypodium* perennials according to their alignment with CoGe (https://genomevolution.org/coge/SynMap.pl), which is based on sequencing data for *B. sylvaticum* (https://genome.jgi.doe.gov/portal/pages/dynamicOrganismDownload.jsf?organism=Bsylvaticum) with reference to their assignment to *B. distachyon* (https://genome.jgi.doe.gov/portal/pages/dynamicOrganismDownload.jsf?organism=BdistachyonBd21_3). For consistency with the Bd and Bs genome designations that were assigned to the annuals *B. distachyon* and *B. stacei* [44], we used the Bp and Bm designations for the *Brachypodium* perennial genomes and *B. mexicanum* genomes, respectively. The chromosomes of the diploid *Brachypodium* perennials and *B. mexicanum* with the same or similar composition of mapped Bd genome-derived BAC clones were given the same chromosome numbers.

### 2.1. Comparative Mapping of the Chromosomes in the Perennial Diploids (2n = 18, 16 Chromosomes)

The arrangement of all the BACs mapped to the chromosomes of the diploids *B. sylvaticum* and *B. pinnatum* (both 2*n* = 18, *x* = 9) is shown in detail on a cytogenetic map (Figure 1). We observed no intraspecific differences in the pattern of clones between the *B. sylvaticum* genotypes PI 297868 and PI 269842 and *B. pinnatum* PI 230113 and PI 345982. Each of the clones hybridized to a single chromosome pair in both species, and their order and arm specificity were the same in the perennial diploid chromosomes and were consistent with their counterparts in the respective reference chromosomes of *B. distachyon*. The clones derived from chromosome Bd1 of *B. distachyon* consistently hybridized to three different chromosomes of *B. sylvaticum* and *B. pinnatum* (2*n* = 18) which were identified as Bp2, Bp6, and Bp7 (Figure 1 and Supplementary Materials Figure S1). The BACs Bd1S/1–5 and Bd1L/24–28 from the distal parts of both chromosome arms of Bd1 mapped consecutively along the entire chromosome Bp2. Clones Bd1S/7–10 and Bd1L/21–23 from the interstitial parts of Bd1 localized along the short and long arm of chromosome Bp6, respectively. The set of BACs Bd1S/11-Bd1L/19 from the central part of Bd1 localized to chromosome Bp7. It is known that chromosome Bd1 of *B. distachyon* arose from two separate nested chromosome fusions (NCF) of three ancestral chromosomes, which are equivalent to the ancestral Os3, Os7, and Os6 “rice-like” chromosomes [4]. Thus, these three ancestral rice chromosome equivalents (ARCEs) correspond to the entire Bp2, Bp6, and Bp7 chromosomes of the diploid *Brachypodium* perennials, respectively (Figure 1).

In the same species, the Bd2-derived clones hybridized to two different chromosomes identified as Bp1 and Bp8 (Figure 1 and Figure S3). The BAC clones Bd2S/2–6 and Bd2L/14–19, from both of the chromosome arms of Bd2 that corresponded to the Os1 ARCE, had an undisrupted linear arrangement along Bp1. The BACs Bd2S/8 to Bd2L/13 from the central part of chromosome Bd2 were localized on chromosome Bp8, which is the Os5 ARCE (Figure 1). These results demonstrate that in the karyotypes of diploid *Brachypodium sylvaticum* and *B. pinnatum*, the homoeologues of Bd2 are represented by two distinct chromosomes, which are equivalent to Os1 and Os5. Comparative mapping with the Bd3-derived BACs revealed two homoeologues, Bp4 and Bp3 (Figure 1 and Figure S4). The BACs Bd3S/1–3 and Bd3L/14–18, from the distal parts of a chromosome of Bd3, were mapped on chromosome Bp4, while the sets Bd3S/4–7 and Bd3L/9–12 from the proximal part of Bd3 localized along chromosome Bp3. Chromosome Bd3 of *B. distachyon* resulted from two separate NCFs of three ARCE—Os2, Os8, and Os10. Comparative mapping indicated that Os2 corresponded to the entire Bp4 and that both Os8 and Os10 corresponded to the Bp3 chromosome of the *Brachypodium* perennials. In the genomes of both diploid 18 chromosome perennials, the full set of Bd4-specific BACs mapped along only one homoeologous counterpart, Bp5 (Figure 1). Probes Bd4S/1–6 and Bd4L/7–13 mapped its entire short and long arm, respectively (Figure S5). All of the applied Bd4-derived probes corresponded to the Os12, Os9, and Os11 ARCEs that were localized together on chromosomes Bp5. Finally, CCB mapping with the Bd5-derived clones revealed their conservative arrangement along one chromosome, which was identified as Bp9. According to these results, the composition of Os12, Os9, and Os11 in the Bp5 and Os4 in the Bp9 chromosome resembled that in Bd4 and Bd5 of *B. distachyon*, respectively (Figure 1).

Interestingly, the karyotypes of some *Brachypodium* diploid perennials consist of only 16 chromosomes. We observed such an atypical, *x* = 8 basic chromosome number in *B. glaucovirens* and in one of the diploid *B. pinnatum* cytotypes (PI 185135). However, barcoding with Bd1–Bd5 chromosome-specific probes showed exactly the same number and position of breakpoint regions as the one in the 18-chromosome diploids. Simultaneous hybridization of Bd1- and Bd3-derived BACs showed that the probes identifying the homoeologues of the Bp3 and Bp6 chromosomes in the 2*n* = 18 chromosome species mapped to the same chromosome pair in the 16 chromosome species (Figure 2 and Figure S8). Such a result clearly indicates the presence of a unique descending dysploidy event via so-called end-to-end fusion (EEF), or a variant mimicking it, of two chromosomes similar to Bp6 and Bp3, resulting in a single chromosome designated Bp6+Bp3. Such convention was applied to all of the other chromosomes with “dual” origin. Among the *Brachypodium* perennial diploids studied to date, such a chromosome has only been found in *B. glaucovirens* and *B. pinnatum* PI 185135 and results in a decrease in their basic chromosome number from *x* = 9 to *x* = 8.

### 2.2. Comparative Mapping of the Chromosomes in the Perennial Allotetraploids (2n = 28 Chromosomes)

The CCB of the allotetraploids *B. pinnatum* and *B. phoenicoides,* both 2*n* = 28 chromosomes, revealed that each single-locus BAC had four hybridization sites that were located on two chromosome pairs. Several genotypes of these polyploids (Table 1) had no intraspecific variation in either the number or the arrangement of the FISH loci (Figures S6 and S7). We were able to distinguish two distinct groups of chromosomes in the karyotypes of these allotetraploids on the basis of their distinctive hybridization signals (Figure 3). One consisted of five pairs of chromosomes and the other nine which can be regarded as subgenomes of Bp with *x* = 5 and *x* = 9.

Although the basic chromosome number of subgenome *x* = 5 is the same as that of genome Bd in *B. distachyon*, CCB with different combinations of the probes from distinct Bd chromosomes shows a unique arrangement of the syntenic segments defined by the ARCE (Figure 3, Figures S6 and S7). Probes Bd1S/1–5 and Bd1L/24–28, which correspond to chromosome Bp2 in perennial diploids, hybridized to the distal parts of the long and short arms of one chromosome in the *x* = 5 subgenome (Figure S2). The central part of this chromosome had hybridization sites of Bd3S/4–7 and Bd3L/9–12 BAC clones, which corresponded to chromosome Bp3 (Figure S4). This indicates a fusion of two ancestral chromosomes that resemble the current Bp2 and Bp3 chromosomes. Based on its BAC clone composition, this chromosome was named Bp2+Bp3 (Figure 3, Figures S6 and S7). Additionally, some of the BAC loci in Bp2+Bp3 had an altered orientation, most likely indicating the presence of a pericentric inversion involving the region delimited by clones Bd3S/4–7 and CEN as well as a paracentric inversion (clones Bd3L/9–12) in the long arm (Figure 3; red arrows). Three other chromosomes in the *x* = 5 subgenome arose as a result of NCFs involving the ARCE, which were similar to those of the Bp *x* = 9 genome. Chromosome Bp4+Bp6 comprised the Bp6 equivalent marked by Bd1S/7–10 and Bd1L/21–23 BAC clones, and the Bp4 equivalent was marked by BACs Bd3S/1–3 and Bd3L/14–18 clones (Figure 3, Figures S2 and S4). Another chromosome was designated Bp5+Bp7 (Figure 3, Figures S6 and S7) as it contained all of the clones from Bd4 that corresponded to both chromosome arms of Bp5 (Figure S5) as well as BACs Bd1S/11–15 and Bd1L/16–19 which marked Bp7 (Figure S2). Heterologous mapping of Bd2-originated probes—BdS2/8–10 and Bd2L/12–13, which in the perennial diploids *x* = 9 corresponded to chromosome Bp8 (Figure S3), and Bd5-derived BACs Bd5S/1 and Bd5L/2–4, which mark chromosome Bp9 (Figure S5)—identified chromosome Bp9+Bp8 (Figure 3, Figures S6 and S7). The arrangement of all of the Bd2S/2–6 and Bd2L/14–19 BAC landmarks in the last chromosome of subgenome *x* = 5 was identical to their distribution along chromosome Bp1 in the perennial diploids. Taking into account the morphological similarity to its counterpart in the *x* = 9 subgenome, this chromosome was also designated Bp1 (Figure 3 and Figure S3).

The BAC–FISH signal distribution in all of the chromosomes belonging to the second group was identical to that found in the chromosomes of the *x* = 9 genome Bp of the *Brachypodium* perennial diploids (2*n* = 18). This observation provided strong evidence that this genome is conserved and constitutes one of the subgenomes of the perennial *Brachypodium* allotetraploids (Figure 1 and Figure 3).

### 2.3. Comparative Chromosome Barcoding of B. mexicanum (2n = 40 Chromosomes)

Each of the Bd-derived clones had four hybridization sites in the chromosome complement of *B. mexicanum* that were usually localized in two morphologically more or less diverse homoeologous chromosome pairs. This implies that *B. mexicanum* is a tetraploid consisting of two 10 chromosome subgenomes (*x* = 10 + 10) which were designated Bm and Bm’. Heterologous mapping of the BACs originating from chromosome Bd1 showed hybridization to seven different chromosomes (Figure 4). The BACs Bd1S/1–6 and Bd1L/24–29, which corresponded to Os3, hybridized to chromosomes Bm2 and Bm2′. However, their exact distribution in these chromosomes was different, suggesting the presence of a large pericentric inversion combined with a duplication of the region that hybridized with the clone Bd1S/1 from chromosome Bm2′ (Figure 4; red arrow). Other BAC clones derived from Bd1 that corresponded to the Os7 ARCE mapped to chromosomes Bm6 and Bm6′. A comparison of the arrangement of clones Bd1L/21–23 indicated the presence of a paracentric inversion in the long arm of Bm6′ (Figure 4; red arrow). The BAC clones that corresponded to the Os6 ARCE were also mapped on individual chromosomes of *B. mexicanum* (i.e., Bm7 and Bm7′) as well as to a short distal segment along Bm3′ (Figures S9 and S10). The CCB of the clones derived from Bd2 highlighted four homoeologues in *B. mexicanum* (Figure 4 and Figure S11). Two of them, Bm1 and Bm1′, carried Bd2S/1–6 and Bd2L/14–20 BAC clone loci corresponding to Os1 ARCE, whereas Os5 ARCE was represented by chromosomes Bm8 and Bm8′ which had the loci of the Bd2S/7–11 and Bd2L/12–13 clones. The Bd3-derived BACs mapped to five homoeologues (Figure 4, Figures S9, S10 and S12). The loci of clones Bd3S/1–3 and Bd3L/13–18 had a similar pattern on both the Bm4 and Bm4′ chromosomes, whereas the probes Bd3S/4–7 and Bd3L/8–12 hybridized to the three homoeologous chromosomes Bm3, Bm3′, and Bm7′. While all of the Bd3-derived BACs mapped to chromosome Bm3, and the clones derived from the short and long arms of Bd3 hybridized separately to Bm3′ and Bm7′, respectively (Figures S9, S10 and S12). Such specific arrangement of the Bd1- and Bd3-derived probes indicates the occurrence of a reciprocal translocation between chromosomes Bm3′ and Bm7′ (Figure 4). The set of clones from Bd4 mapped to four different chromosomes of *B. mexicanum*. The BACs Bd4S/1–3 and Bd4L/8–13, which correspond to the Os12+Os9 ARCE segments in Bd4, spanned the entire length of the short and long arms of chromosome Bm5, respectively. Another homoeologue, Bm5′, had a similar distribution of these clones except for the terminal fragment, which contained the clones Bd4L/12–13 that underwent an inter-arm translocation that was connected with the inversion (Figure 4; red arrows). Chromosome Bm5 was the only 35S rDNA-bearing chromosome with a distinct secondary constriction on its short arm, whereas Bm5′ contained a 5S rDNA site that was localized subterminally on the long arm. Clones Bd4S/4–6 and Bd4L/7, which in Bd4 are associated with the Os11 ARCE, hybridized with chromosomes Bm10 and Bm10′ (Figure S13). These BACs mapped in a conserved order along Bm10 as in Bd4, but the Bm10′ hybridization sites of Bd4L/7 and 5S rDNA were located together on the long arm with clones Bd4S/4–6. This suggests the presence of a small pericentric inversion that involved the proximal region of Bm10′ (Figure 4; red arrows). Bd5-derived BACs hybridized with two chromosomes, Bm9 and Bm9′, in the same manner as in the Bd genome (Figure 4 and Figure S14).

## 3. Discussion

### 3.1. Karyotype Evolution in the Perennial Diploids

Most of the *Brachypodium* species in this group have *x* = 9 chromosomes which suggests that some chromosome fusions must have occurred during the divergence of their karyotypes from the 12 chromosome Intermediate Ancestral Grass Karyotype (IAGK) [23]. We showed the same distribution pattern of BAC–FISH signals in *B. sylvaticum* and *B. pinnatum* (2*n* = 18) chromosomes (Figure 1). Their karyotypes had the same structure and pattern of dysploidy events. Chromosomes Bp3 and Bp5 were formed by NCFs, which involved Os8+Os10 and Os12+Os9+Os11, respectively. All of the seven remaining chromosomes (i.e., Bp2, Bp6, Bp7, Bp1, Bp8, Bp4, and Bp9) did not undergo NCF events and directly correspond to Os3, Os7, Os6, Os1, Os5, Os2, and Os4 ARCEs, respectively. The same Os12+Os9+Os11 fusions as in Bp5 were observed in Bd4 of the reference *B. distachyon* karyotype, whereas, in Bp3, only one NCF (Os8+Os10) was detected. This particular fusion was also present in chromosome Bs3 of the annual *B. stacei* and its allotetraploid derivative *B. hybridum* [44].

**Figure 5:**
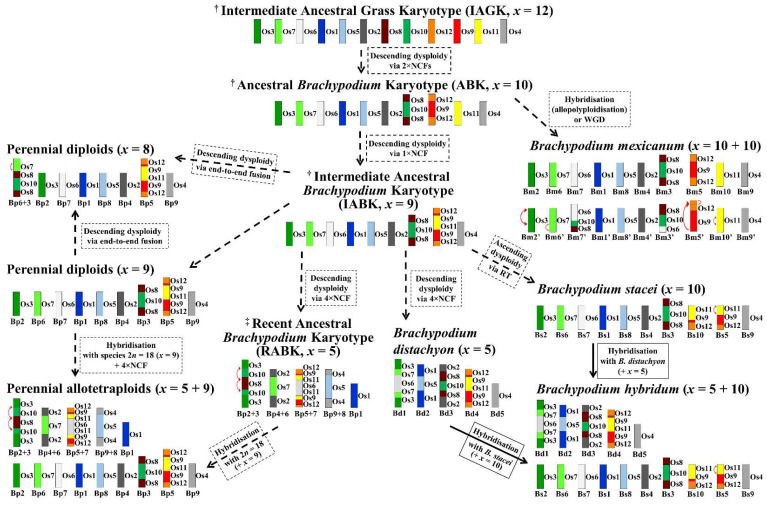
A comprehensive model of karyotype evolution in the genus *Brachypodium* inferred from the Intermediate Ancestral Grass Karyotype based on IBI [4] and Murat et al. [22]. The model is based on the results of the comparative chromosome barcoding-based mapping in all of the *Brachypodium* perennial diploids, allotetraploids, and in the *B. mexicanum* that were analyzed in this study as well as in the *Brachypodium* annuals, *B. stacei* and *B. hybridum* [44]. Os—ancestral rice chromosome equivalents (ARCEs). Genome/subgenome designations: Bd—*B. distachyon*, Bp—*Brachypodium* perennials, Bm, Bm’—*B. mexicanum*, Bs—*B. stacei*. Dashed arrows indicate hypothetical evolutionary pathways of *Brachypodium* karyotypes. In some cases, two alternative pathways are shown. The solid arrow shows the origin of *B. hybridum* which is experimentally determined [49]. Red arrows point to the minor intrachromosomal rearrangements (inversions, translocations). ^†^ Species with such karyotypes are extinct. ^‡^ Diploid species with this karyotype are extinct or unknown.

Moreover, it was also found in all homoeologues across the *Brachypodium* species, which suggests that it might be one of the most ancient NCF events involving two ancestral chromosomes that were fused in the putative *x* = 10 Ancestral *Brachypodium* Karyotype (ABK, Figure 5). In the perennial diploids, Os12, Os9, and Os11 comprised chromosome Bp5 (Figure 1) and in *B. distachyon* chromosome Bd4, while, in *B. stacei*, they were found in two chromosomes (i.e., Bs10 and Bs5) [44]. Thus, it can be inferred that the Os12+Os9+Os11 fusions occurred before the divergence of *B. stacei* (16.2 Ma), *B. distachyon* (10.6 Ma), and the core perennial clade (6.1 Ma) [6,8,13,15]. As was shown by Lusinska et al. [44], the Bs10 and Bs5 split was possibly the result of a Robertsonian rearrangement, which was responsible for an ascending dysploidy (Figure 5) in the Bs genome.

Most of the perennial diploids had 2*n* = 18 chromosomes but species with 2*n* = 16 have also been described. We revealed that, in *B. glaucovirens* and in *B. pinnatum* PI 185135, a combination of the Bd1- and Bd3-derived BAC-based probes hybridized to the same chromosome, indicating the presence of an EEF (or asymmetric reciprocal translocation between the ends of two metacentric chromosomes that mimics EEF) involving chromosomes similar to Bp6 and Bp3 which is responsible for the descending dysploidy from *x* = 9 to *x* = 8 via the formation of a unique chromosome, Bp6+Bp3, in this karyotype (Figure 2). Based on nuclear genome size estimates [12], it can be assumed that this dysploidy was not associated with genome downsizing. When two (sub)metacentric chromosomes are involved in EEF, at least one becomes telo- or acrocentric via a pericentric inversion [46]. Such an inversion was detected in the chromosome Bp6+Bp3, and it can be assumed that this rearrangement occurred in an ancestral chromosome that was similar to Bp6 before its EEF with Bp3. Pericentric inversions that accompany chromosome fusions were detected on the dense genetic and cytogenetic maps of some Brassicaceae [39,47] and *Cucumis* [41] representatives. In grasses, the occurrence of EEFs was reported in maize and Wang et al. [48] postulated that such a mechanism was responsible for the organization of several chromosomes. However, EEFs seem to be rare in *Brachypodium*.

### 3.2. Karyotype Evolution in the Perennial Polyploids

It is recognized that polyploidy followed by subsequent diploidization are major mechanisms that drive genomic diversity and evolution in angiosperms [22,50]. Current comparative karyotypic data suggest that the post-polyploid descending dysploidies are more common than the ascending ones. A return to a reduced diploid state usually occurs via reciprocal translocations, which are either NCFs that commonly occur in grasses [4,44,51,52] and some eudicots [40,41,53] or EEFs which seem to be common in eudicots [39,50,54].

The allotetraploid nature of many *Brachypodium* perennials has already been inferred from comparative genomic in situ hybridization [12] and CCP-based analyses [9]. Previous studies suggested that the 28 chromosome *Brachypodium* species can be allopolyploids, which may have been derived from diploid 2*n* = 18 (some genotypes of *B. sylvaticum* and *B. pinnatum*) and 2*n* = 10 (*B. distachyon*) progenitors [9,10,12]. However, the results of other cytomolecular analyses [14,55] and recent phylogenetic studies [13] suggested that the perennial allopolyploids had originated from the hybridizations of various 2*n* = 18 core-perennial diploids. In this study, we confirmed the allopolyploid nature of *B. phoenicoides* and *B. pinnatum* (2*n* = 28) and identified all of the chromosomes that had been derived from putative parental genomes (Figure 3). Those of the *x* = 9 subgenome corresponded to the nine Bp chromosomes that are found in the perennial diploids, whereas five chromosomes that belong to the second subgenome were characterized by a plethora of complex descending dysploidy events. We showed that three ancestral NCFs involving five ancestral chromosomes (Os8+Os10 and Os12+Os9+Os11) are present in the genomes of *Brachypodium* annuals [44] as well as in all *Brachypodium* perennials except *B. mexicanum*. Moreover, four additional NCFs were found in the subgenome *x* = 5 of *Brachypodium* perennial allotetraploids. These involved eight ancestral chromosomes and are probably more recent, since their patterns did not reflect any of the several rounds of descending dysploidy events known for *B. distachyon* chromosomes (Figure 3). Recently, an inferred homology among Triticeae, rice, and *B. distachyon* chromosomes revealed different chromosome evolution trajectories in the Triticeae and *B. distachyon* lineages. Seven Triticeae chromosomes resulted from four NCFs and one EEF of 12 ARCEs that constitute the IAGK, while five *B. distachyon* chromosomes arose through seven NCF events. Interestingly, neither a single fusion event that formed intermediate and/or extant chromosomes was shared by the Triticeae and *B. distachyon* lineages [56] nor by Triticeae and *Brachypodium* perennial allotetraploids in this study.

Initially, the basic chromosome number of *B. mexicanum* was suggested as five [16], but the results of more recent studies estimated it to be ten and indicated a possible allotetraploid nature of this species [8,9]. Our current study provides a strong indication that *B. mexicanum* is a tetraploid with a karyotype consisting of two subgenomes with *x* = 10 in each (Figure 4). Their individual Bm and Bm’ homoeologues display various degrees of similarity with several peri- and paracentric inversions and translocations identified in some of the homoeologues (Figure 4). Because of the similarity of its subgenomes, it is not clear if *B. mexicanum* is an allotetraploid or autotetraploid with structural changes in the Bm’ subgenome after a WGD. In contrast to other *Brachypodium* representatives, we revealed that *B. mexicanum* chromosomes carry only two ancient fusions, which is the lowest number within the genus to date. The first was an Os8+Os10 fusion, which was present in chromosomes Bm3, Bm3′, and Bm7′. The second fusion was the Os12+Os9 that was found in chromosomes Bm5 and Bm5′ (Figure 4). The Os8+Os10 and Os12+Os9 fusions were present in all of the *Brachypodium* species studied to date. Moreover, *B. mexicanum* is the only *Brachypodium* representative that does not have Os12+Os9 ARCEs fused with Os11.

### 3.3. Brachypodium Karyotype Evolution

The current study together with cytomolecular analyses of *Brachypodium* annuals [44] permitted the creation of a hypothetical model of *Brachypodium* karyotype evolution (Figure 5). It begins with the IAGK (*x* = 12) through separate descending dysploidy events which resulted in inferred putative ABK with *x* = 10 and an Intermediate Ancestral *Brachypodium* Karyotype (IABK) with *x* = 9 chromosomes. Based on the results of cytomolecular mapping, we deduced that most of the perennial and annual species probably evolved from an ancestor that had IABK, because of the presence of the Os12, Os9, and Os11 segments that were already fused in their genomes. We inferred that *B. mexicanum* evolved directly from an *x* = 10 ancestor via autopolyploidization or allopolyploidization, which is evidenced by the lack of Os12+Os9 fused with Os11. The phylogenetic analysis of plastid and nuclear loci suggest the existence of an ancestral homoeologous subgenome not found in current diploid species and present only in *B. mexicanum* and the high polyploids (*B. boissieri*, *B. retusum*) [8,13]. This early split was followed by the split of diploid *B. stacei* and its close polyploid subgenomes, such as the one present in *B. mexicanum*, and the split of a more recent sister relation of diploid *B. distachyon* and the core perennial clade composed of diploid and polyploid species [8,13]. These phylogenetic data partially corroborate our findings in *B. mexicanum,* insofar as the first lineage that diverged from a common ancestor was characterized by *x* = 10. However, the arrangement of the fused Os12+Os9 and the separate Os11 in *B. mexicanum* seems to be in agreement with some of the phylogenetic data of Díaz-Pérez et al. [13] that indicated the involvement of an ancestral genome older than that of *B. stacei* in *B. mexicanum*. However, this conclusion is confounded by other data that identifies an additional subgenome homoeologous to that of *B. stacei.* Thus, the results of CCB clearly contradict the notion that one of the *B. mexicanum* subgenomes originated from the genome Bs, but this assumption is based only on the karyotypic data (Figure 5) [44]. The cytomolecular data also support the separate evolution of the diploid annuals from the ancestor with IABK (*x* = 9). We assume that the divergence of genome Bs could occur via ascending dysploidy and that the genome Bd could emerge via multiple descending dysploidy events (Figure 5) [44], though the evolutionary timing of these events could not be established from the current data.

The lineages of the extant *Brachypodium* perennial diploids (2*n* = 18) are likely to have originated from an ancestor that was characterized by *x* = 9 IABK. Unlike genome Bd, they did not undergo the series of NCFs that was responsible for the chromosome number reduction in *B. distachyon*. However, the *x* = 8 chromosome genomes Bp of *B. glaucovirens* and *B. pinnatum* PI 185135 might have arisen either directly from an *x* = 9 intermediate ancestor with IABK or from other perennial diploids via the occurrence of one EEF event resulting in the chromosome Bp6+Bp3 (Figure 5). The current data support two hypothetical models of the origin of the perennial allotetraploids. One infers the existence of a progenitor species with a Recent Ancestral *Brachypodium* Karyotype (RABK) consisting of five chromosomes (Figure 5) that contributed to the cross with the *x* = 9 diploids followed by genome doubling. This model explains well the striking conservation of the NCF patterns that are observed in the subgenome Bp *x* = 5 in various perennial allotetraploids. However, the existence of a RABK *x* = 5 progenitor is speculative. It cannot be ruled out that the NCFs that were observed in the chromosomes of the Bp *x* = 5 subgenome reflect a putative “ghost” genome which is now extinct from or unknown in the diploids.

The other model assumes that perennial allotetraploids resulted from the hybridization of two different *x* = 9 diploids followed by descending dysploidy via four NCFs (Figure 5) which was also postulated by Catalan et al. [8]. Considering this hypothetical pathway, it is likely that the descending dysploidy involves only one of the contributing ancestral genomes. This conclusion is supported by the fact that the inter-chromosomal fusions never involved the same ARCE. The NCF patterns that are specific for the subgenome Bp *x* = 5 chromosomes were highly conserved in several genotypes of both the *B. phoenicoides* and *B. pinnatum* allotetraploids (Figures S6 and S7). This implies that the genomic and possibly taxonomic variability between these taxa might be the result of their independent divergence occurring after polyploidization.

Recent phylogenetic data discriminate between homoeologous “ancestral” and “recently evolved” gene copies at the GIGANTEA locus and to a lesser extent also within the ITS and ETS of ribosomal DNA loci thus providing new insight into the origin of perennial allopolyploids [8,13]. Phylogenetic analyses indicated the presence of genome donors in *B. phoenicoides* that are homoeologous to *B. pinnatum* 2*n* = 18 and *B. sylvaticum*. However, the genomic composition of *B. pinnatum* 2*n* = 28 is still not fully resolved since its different cytotypes have alleles that are associated with the core genomes homoeologous to those of *B. glaucovirens, B. sylvaticum* and *B. arbuscula* [13]. Based on these findings, the assumption is that only the perennial genomes formed the allotetraploids *B. pinnatum* and *B. phoenicoides*, which contradicts our earlier hypothesis that *B. distachyon* is also one of the genome donors [9,12]. However, the CCB-based findings of the present study clearly indicate the contribution of an unknown Bp subgenome *x* = 5 that shares the same chromosome number but has a completely different syntenic segment composition of all of its chromosomes compared to Bd. This enables the complex karyotype organization in the perennial allotetraploids to be resolved.

## 4. Materials and Methods

### 4.1. Plant Material

Six diploid and six allopolyploid genotypes of five perennial *Brachypodium* species were used in this study with reference to the *B. distachyon* inbred line Bd21. Information about their origin and basic cytogenetic properties is provided in Table 1. 

### 4.2. Chromosome Preparation

The multi-substrate chromosome preparations were made according to the protocols of Hasterok et al. [57] and Jenkins and Hasterok [37]. In brief, young seedlings were incubated for 24 h in a box of ice, then fixed for several hours in 3:1 (*v*/*v*) methanol/glacial acetic acid and stored at −20 °C until they were used. Excised root tips were digested in an enzyme mixture containing 8% (*v/v*) pectinase, 1% (*w/v*) cellulase (Sigma–Aldrich, St. Louis, MO, USA), and 1% (*w/v*) cellulase, “Onozuka R-10” (Serva, Heidelberg, Germany), for 2 h at 37 °C for all species except *B. mexicanum*, where these enzymes were used at concentrations of 8%, 0.5%, and 0.5%, respectively. For squashed chromosome preparations, the meristems of each species were dissected in a small volume of 45% acetic acid followed by a separate mounting of the digested material on a slide. 

### 4.3. Probe Labelling and FISH

The BAC clones that were used in this study (Table S1) originated from the BD_ABa and BD_CBa genomic DNA libraries and were derived from the FingerPrinted Contigs that had been assigned to the respective reference chromosomes of *B. distachyon* [38]. The details regarding centromeric clone BD_CBa0033J12 and the selection of the low-repeat BAC clones are described in Lusinska et al. [44]. Each clone was mapped to chromosome preparations of several individuals of each species or accession in order to gauge intraspecific variation (Table 1). 

The BAC DNA was isolated using the standard alkaline lysis method and then labelled by nick-translation with tetramethylrhodamine-5-dUTP by nick-translation (Roche, Basel, Switzerland) with digoxigenin-11-dUTP or biotin (both Roche). The nick-translated 25S and 5S ribosomal DNA probes were based on a clone that contained a 2.3 kb *Cla*I fragment of the 25S rRNA gene of *A*. *thaliana* [58] and on a pTa794 clone that contained the 5S rRNA gene from common wheat [59], respectively. The probe labelling and FISH followed the Jenkins and Hasterok [37] protocol with a minor modification by Lusinska et al. [44]. All of the images were acquired using an AxioCam Mrm high-sensitivity monochromatic camera attached to an AxioImager.Z.2 wide-field epifluorescence microscope (both Zeiss, Oberkochen, Germany) and processed uniformly using ZEN 2.3 Pro (Zeiss) and Photoshop CS3 (Adobe, San Jose, CA, USA).

## 5. Conclusions

Our current analyses in several *Brachypodium* species enabled the dissection of their karyotype organization, tracking of the evolutionary histories of individual chromosomes, and the identification of an additional *x* = 5 genome (RABK, *x* = 5). It contributed a subgenome to the perennial allotetraploids, such as *B. pinnatum* 2*n* = 28 and *B. phoenicoides,* and is probably now extinct in diploids. It seems that NCFs are much more common players than EEFs in the descending dysploidy in *Brachypodium* genomes, although one such rare EEF event is responsible for the difference in chromosome number between the 2*n* = 18 and 2*n* = 16 perennial diploids. Interestingly, all perennials lack the split of a Bd4-like chromosome that causes the ascending dysploidy from *x* = 9 to *x* = 10 which is found in all annuals except *B. distachyon*. Thus, it seems that this structural event is exclusive to the genome Bs. Although our study offers significant insight into the organization of the *B. mexicanum* karyotype and places its subgenomes among the first to have diverged from ABK with *x* = 10, it does not provide a definite answer as to whether this species is of an allopolyploid origin or whether it represents a highly restructured autopolyploid. The findings of this study enabled us to propose a model of the karyotype evolution in the *Brachypodium* genus that is inferred from IAGK *x* = 12 and to provide the most comprehensive view on the organization of *Brachypodium* genomes at the chromosomal level to date.

## Figures and Tables

**Table 1:** General characteristics of the *Brachypodium* species that were used in this study.

Species	Accession Number	2*n*	*x*	Ploidy Level	Origin	Source *
*B. distachyon*	Bd21	10	5	2×	Iraq	USA
*B. sylvaticum*	PI 297868	18	9	2×	Australia	USA
	PI 269842	18	9	2×	Tunisia	USA
*B. glaucovirens*	PI 4202	16	8	2×	Greece, Crete	Germany
*B. pinnatum*	PI 185135	16	8	2×	Iraq	USA
	PI 230113	18	9	2×	Iran	USA
	PI 345982	18	9	2×	Norway	USA
	PI 249722	28	5 + 9	4×	Greece	USA
	PI 251445	28	5 + 9	4×	Turkey	USA
	PI 430277	28	5 + 9	4×	Ireland	USA
*B. phoenicoides*	PI 253503	28	5 + 9	4×	Spain	USA
	PI 89817	28	5 + 9	4×	Spain	USA
*B. mexicanum*	Bmex347	40	10 + 10	4×	Mexico	UK

* USA: United States Department of Agriculture—National Plant Germplasm System, Beltsville, MD; Germany: Botanical Garden Berlin-Dahlem; UK: University of Leicester, Leicester (from Clive A. Stace); PI: Plant introduction.

## Supplementary Materials

Supplementary materials can be found at https://www.mdpi.com/1422-0067/20/22/5557/s1.

## Abbreviations

ABK	Ancestral *Brachypodium* Karyotype (*x* = 10)
ARCE	Ancestral rice chromosome equivalent
Bd	Chromosome complement of *B. distachyon* (*x* = 5)
Bp	Chromosome complement(s) of *Brachypodium* perennials (*x* = 9, 8, and 5)
Bm (Bm’)	Chromosome complement(s) of *B. mexicanum* (*x* = 10 + 10)
Bs	Chromosome complement of *B. stacei* (*x* = 10)
BAC	Bacterial artificial chromosome
CCB	Comparative chromosome barcoding
CCP	Comparative chromosome painting
CEN	Centromeric BAC BD_CBa0033J12
EEF	End-to-end fusion
FISH	Fluorescence in situ hybridization
IABK	Intermediate Ancestral *Brachypodium* Karyotype (*x* = 9)
IAGK	Intermediate Ancestral Grass Karyotype (*x* = 12)
Ma	Million years
NCF	Nested chromosome fusion
RABK	Recent Ancestral *Brachypodium* Karyotype (*x* = 5)
WGD	Whole genome duplication
