# Supplementary material for: Comparatively Barcoded Chromosomes of Brachypodium Perennials Tell the Story of Their Karyotype Structure and Evolution
Source: Int J Mol Sci. 2019 Nov 7;20(22):5557. doi: 10.3390/ijms20225557 (PMC6888173; doi:10.3390/ijms20225557)
Supplement: Supplementary file 1 [file ijms-20-05557-s001.pdf]

International Journal of
Molecular Sciences

MDPI

Supplementary Materials

Article Title: Comparatively Barcoded Chromosomes of *Brachypodium* Perennials Tell the Story of Their Karyotype Structure and Evolution

Authors: Joanna Lusinska, Alexander Betekhtin, Diana Lopez-Alvarez, Pilar Catalan, Glyn Jenkins, Elzbieta Wolny, and Robert Hasterok

The following Supplementary Materials are available for this article:

Supplementary Tables

Table S1. Specification of the bacterial artificial chromosome (BAC) clones that were used for the fluorescence in situ hybridization-based comparative chromosome barcoding.

BAC Name	BAC Clone Identifier *	Position in the Genome (bp)
CEN	BD_CBa0033J12	-
Bd1S/1	BD_CBa0027N17	Bd1: 560624 : 710332
Bd1S/2	BD_ABa0004B12	Bd1: 3276891 : 3460444
Bd1S/3	BD_ABa0017K22	Bd1: 5375697 : 5509098
Bd1S/4	BD_CBa0030L10	Bd1: 8680898 : 8845282
Bd1S/5	BD_ABa0032D10	Bd1: 9006125 : 9148678
Bd1S/6	BD_ABa0027D04	Bd1: 12706461 : 12847057
Bd1S/7	BD_ABa0020A04	Bd1: 15092918 : 15238493
Bd1S/8	BD_ABa0009N18	Bd1: 17150298 : 17335777
Bd1S/9	BD_CBa0002O16	Bd1: 20013520 : 20160236
Bd1S/10	BD_CBa0022H13	Bd1: 23114454 : 23242441
Bd1S/11	BD_ABa0018O15	Bd1: 25727688 : 25878318
Bd1S/12	BD_ABa0044I06	Bd1: 28135872 : 28292480
Bd1S/13	BD_ABa0036J15	Bd1: 31222238 : 31387974
Bd1S/14	BD_CBa0024I19	Bd1: 32507293 : 32633286
Bd1S/15	BD_ABa0004L01	Bd1: 34316249 : 34466638
Bd1L/16	BD_ABa0002I22	Bd1: 39952805 : 39424325
Bd1L/17	BD_CBa0004P09	Bd1: 43536825 : 43670757
Bd1L/18	BD_CBa0025P22	Bd1: 46564769 : 46692954
Bd1L/19	BD_CBa0044L08	Bd1: 48612347 : 48783561
Bd1L/20	BD_ABa0003G14	Bd1: 50139085 : 50274374
Bd1L/21	BD_CBa0035K24	Bd1: 51720482 : 51914140
Bd1L/22	BD_ABa0046G17	Bd1: 54082210 : 54253048
Bd1L/23	BD_CBa0028P17	Bd1: 57093738 : 57225377
Bd1L/24	BD_ABa0010A14	Bd1: 59503419 : 59676696
Bd1L/25	BD_Aba0034M17	Bd1: 62501248 : 62642447
Bd1L/26	BD_ABa0013D23	Bd1: 64120769 : 64297730
Bd1L/27	BD_ABa0019B19	Bd1: 67392232 : 67529032
Bd1L/28	BD_ABa0043A05	Bd1: 68898017 : 69053532
Bd1L/29	BD_ABa0033F06	Bd1: 74020535 : 74180685
Bd2S/1	BD_ABa0038A01	Bd2: 1022 : 132144
Bd2S/2	BD_ABa0027K15	Bd2: 1864643 : 2004976
Bd2S/3	BD_ABa0028O04	Bd2: 3492740 : 3587755
Bd2S/4	BD_ABa0045F24	Bd2: 6004397 : 6146555
Bd2S/5	BD_ABa0012B07	Bd2: 9006125 : 9148678
Bd2S/6	BD_ABa0005E09	Bd2: 10380990 : 10507985
Bd2S/7	BD_CBa0023P23	Bd2: 10380990 : 10507985
Bd2S/8	BD_ABa0044D02	Bd2: 14006553 : 14195269
Bd2S/9	BD_ABa0047D12	Bd2: 17856422 : 17996794

BAC Name	BAC Clone Identifier *	Position in the Genome (bp)
Bd2S/10	BD_ABa0026K14	Bd2: 19861012 : 20005795
Bd2S/11	BD_CBa0038L02	Bd2: 22509927 : 22639901
Bd2L/12	BD_ABa0014K11	Bd2: 34309867 : 34503922
Bd2L/13	BD_CBa0022I07	Bd2: 38509106 : 38646001
Bd2L/14	BD_ABa0008H07	Bd2: 42500887 : 42664133
Bd2L/15	BD_CBa0041G17	Bd2: 44876290 : 45007631
Bd2L/16	BD_CBa0031I09	Bd2: 46500135 : 46639653
Bd2L/17	BD_CBa0038L04	Bd2: 50005019 : 50143082
Bd2L/18	BD_CBa0036G07	Bd2: 53816466 : 54010118
Bd2L/19	BD_CBa0047O03	Bd2: 55698147 : 56502216
Bd2L/20	BD_ABa0038G14	Bd2: 57002804 : 57148130
Bd3S/1	BD_CBa0028O16	Bd3: 856255 : 1007650
Bd3S/2	BD_ABa0015A18	Bd3: 4001904 : 4157452
Bd3S/3	BD_ABa0018B12	Bd3: 6003300 : 6153924
Bd3S/4	BD_CBa0016A22	Bd3: 11505050 : 11712720
Bd3S/5	BD_CBa0010J18	Bd3: 13993335 : 14131952
Bd3S/6	BD_ABa0022G01	Bd3: 16038657 : 16055486
Bd3S/7	BD_ABa0033D16	Bd3: 22106200 : 22299788
Bd3L/8	BD_ABa0036L01	Bd3: 31849467 : 32007174
Bd3L/9	BD_CBa0011M04	Bd3: 36854229 : 37002472
Bd3L/10	BD_ABa0037F23	Bd3: 39500638 : 39639896
Bd3L/11	BD_ABa0013E06	Bd3: 42292206 : 42500698
Bd3L/12	BD_ABa0038N13	Bd3: 44001051 : 44142538
Bd3L/13	BD_CBa0040H10	Bd3: 46810232 : 47007666
Bd3L/14	BD_ABa0026M18	Bd3: 49347850 : 49503810
Bd3L/15	BD_ABa0037F15	Bd3: 50854746 : 51004145
Bd3L/16	BD_ABa0037C10	Bd3: 52501229 : 52687354
Bd3L/17	BD_ABa0008G22	Bd3: 55503533 : 55665230
Bd3L/18	BD_ABa0020N10	Bd3: 57504387 : 57653389
Bd4S/1	BD_CBa0030B12	Bd4: 2007584 : 2157984
Bd4S/2	BD_ABa0021K11	Bd4: 5356098 : 5506730
Bd4S/3	BD_CBa0040J03	Bd4: 7830905 : 8001843
Bd4S/4	BD_CBa0021B09	Bd4: 9502901 : 9667864
Bd4S/5	BD_ABa0043D11	Bd4: 11006774 : 11150531
Bd4L/6	BD_ABa0010I18	Bd4: 14002249 : 14164264
Bd4L/7	BD_ABa0006J17	Bd4: 29358544 : 29516826
Bd4L/8	BD_ABa0020D08	Bd4: 32504625 : 32642850
Bd4L/9	BD_CBa0035E05	Bd4: 39350118 : 39526113
Bd4L/10	BD_ABa0003H15	Bd4: 40835257: 41003446
Bd4L/11	BD_CBa0038H23	Bd4: 42789149 : 43003220
Bd4L/12	BD_ABa0043N14	Bd4: 45504848 : 45661980
Bd4L/13	BD_ABa0041I03	Bd4: 48350055 : 48507632
Bd5S/1	BD_ABa0019O20	Bd5: 1091367 : 1236179
Bd5L/2	BD_ABa0045F23	Bd5: 13499779 : 13653343
Bd5L/3	BD_ABa0023L21	Bd5: 17634500 : 17679830
Bd5L/4	BD_CBa0024J19	Bd5: 20845837 : 21003148
Bd5L/5	BD_CBa0032J06	Bd5: 23870997 : 24003288
Bd5L/6	BD_ABa0019J13	Bd5: 25906054: 26098440

* More detail can be found in the NCBI database under the following URLs: http://www.ncbi.nlm.nih.gov/clone/library/genomic/424 (BD_ABa library) and http://www.ncbi.nlm.nih.gov/clone/library/genomic/426/ (BD_CBa library).

Supplementary Figures

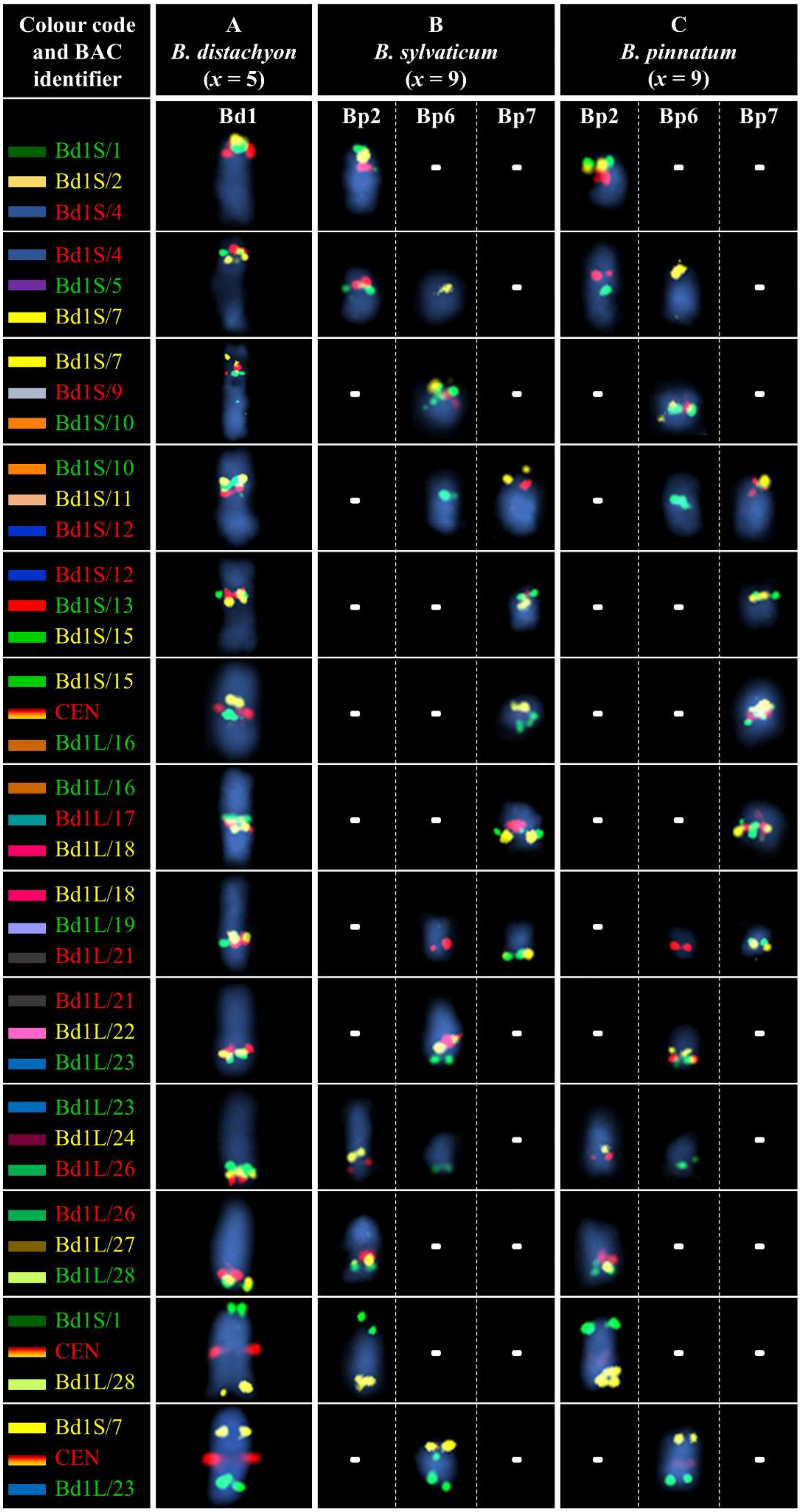

Figure S1. The BAC-FISH-based comparative chromosome barcoding with the clones derived from chromosome Bd1 of (**A**) *Brachypodium distachyon* ($2n = 10$, $x = 5$) mapped to chromosomes Bp2, Bp6, and Bp7 of the diploids (**B**) *B. sylvaticum* and (**C**) *B. pinnatum* (both $2n = 18$, $x = 9$). Only one homologue from a pair is shown. The colors of the BAC identifiers in the first column indicate the fluorochrome that was used (green, FITC; red, tetramethylrhodamine; yellow [false color], Alexa Fluor 647). The chromosomes were counterstained with DAPI (blue). The colored bars on the left and the BAC identifiers that were assigned to specific clones correspond to those on the cytogenetic maps in Figure 1. The BACs Bd1S/1–5 and Bd1L/24–28 from Bd1 mapped to chromosome Bp2, probes Bd1S/7–10 and Bd1L/21–23 hybridized to Bp6 and probes Bd1S/11–Bd1L/19 with Bp7. The probes 1S/4–7, 1S/10–12, 1L/18–19–21, and 1L/23–24–26 showed chromosomal breakpoints in the genome Bp compared to the chromosomal fusion points in the genome Bd. Probes Bd1S/1 + CEN + Bd1L/28 map to chromosome Bp2, whereas probes Bd1S/7 + CEN + Bd1L/23 hybridized to chromosome Bp6 and Bd1S/15 + CEN + Bd1L/16 with chromosome Bp7 thus indicating the presence of two NCF events in the Bd genome of *B. distachyon* that involve three ancestral chromosomes which were similar to Bp2, Bp6, and Bp7 of the $x = 9$ genome Bp.

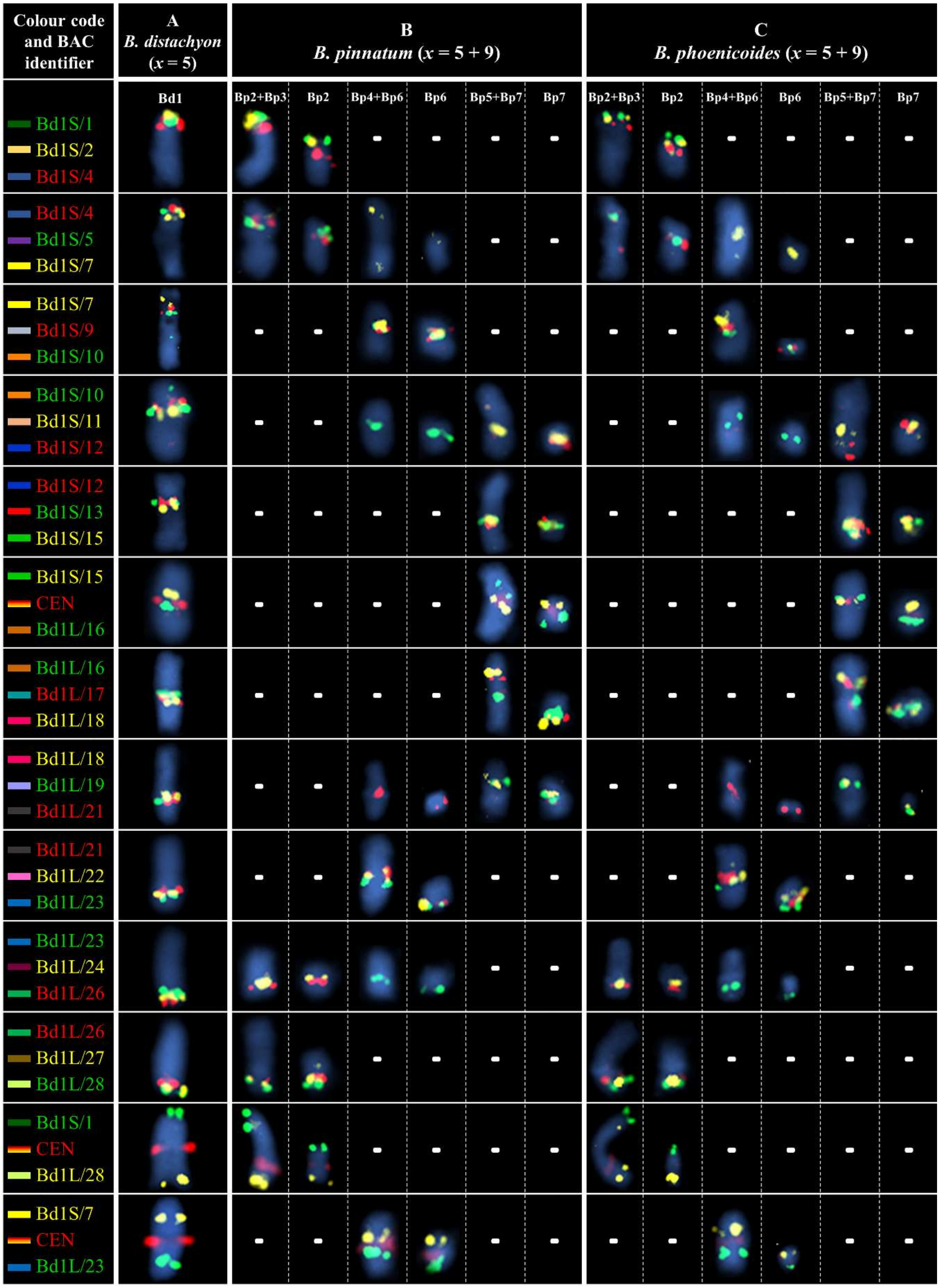

Figure S2. The BAC–FISH-based comparative chromosome barcoding with the clones derived from chromosome Bd1 of (**A**) *Brachypodium distachyon* (2*n* = 10, *x* = 5) mapped to chromosomes Bp2+Bp3, Bp2, Bp4+Bp6, Bp6, Bp5+Bp7, and Bp7 of the allotetraploids (**B**) *B. pinnatum* and (**C**) *B. phoenicoides* (both 2*n* = 28, *x* = 5 + 9). Only one homologue from a pair is shown. The colors of the BAC identifiers in the first column indicate the fluorochrome that was used (green, FITC; red, tetramethylrhodamine; yellow [false color], Alexa Fluor 647). The chromosomes were counterstained with DAPI (blue). The

colored bars on the left and the BAC identifiers that were assigned to specific clones correspond to those on the cytogenetic maps in Figure 3. BACs Bd1S/1–5 and Bd1L/24–28 from Bd1 mapped to chromosomes Bp2+Bp3 and Bp2, the probes Bd1S/7–10 and Bd1L/21–23 to Bp4+Bp6 and Bp6, and the probes Bd1S/11–Bd1L/19 to Bp5+Bp7 and Bp7. The probes 1S/4–7, 1S/10–12 and 1L/18–21, and 1L/23–26 show the chromosomal breakpoints in both Bp subgenomes compared to the chromosomal fusion points in genome Bd. Probes Bd1S/1 + CEN + Bd1L/28 map to chromosomes Bp2+Bp3 and Bp2, whereas probes Bd1S/7 + CEN + Bd1L/23 hybridized to chromosomes Bp4+Bp6, Bp6, and Bd1S/15 + CEN + Bd1L/16 with chromosomes Bp5+Bp7 and Bp7 thus indicating the presence of two NCF events in the Bd genome of *B. distachyon* that involved three ancestral chromosomes, which were similar to Bp2, Bp6, and Bp7 of the $x = 9$ genome/subgenome Bp.

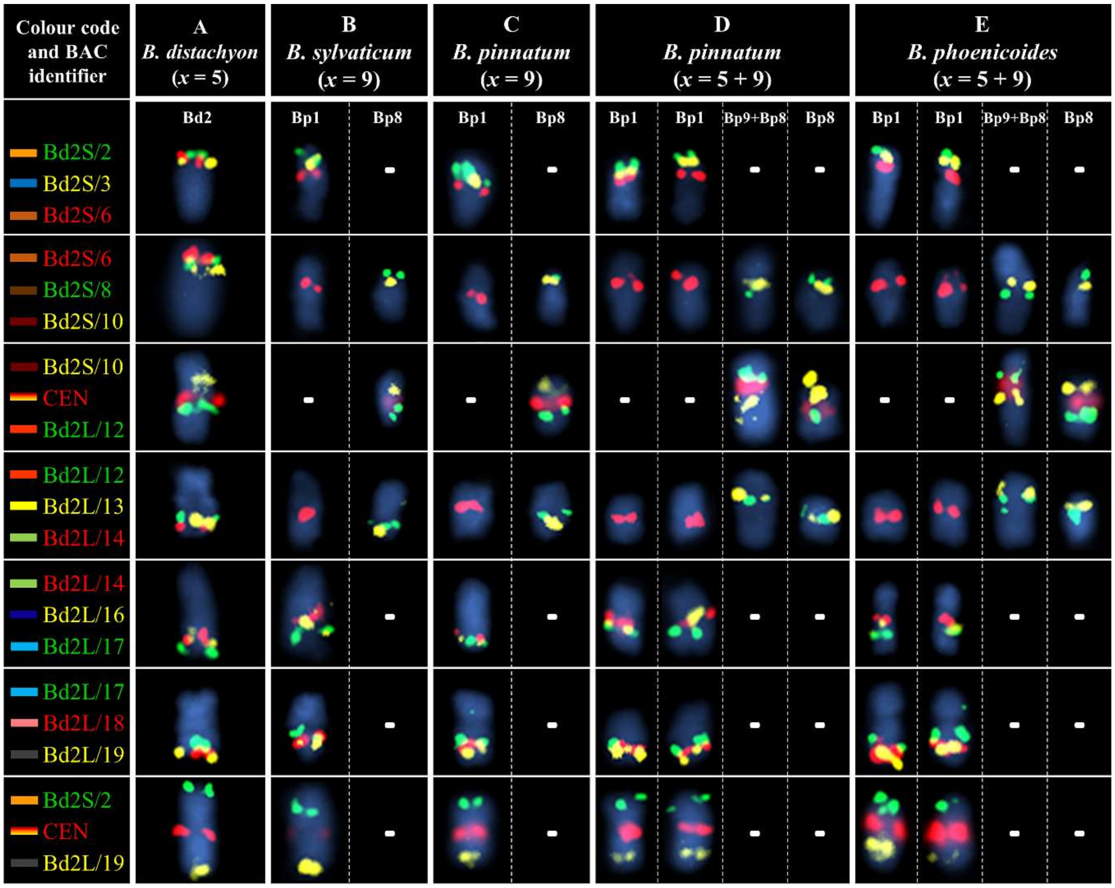

Figure S3. The BAC–FISH-based comparative chromosome barcoding with the clones derived from chromosome Bd2 of (**A**) *Brachypodium distachyon* (2*n* = 10, *x* = 5) mapped to chromosomes Bp1 and Bp8 of the diploids (**B**) *B. sylvaticum* and (**C**) *B. pinnatum* (both 2*n* = 18, *x* = 9) and chromosomes Bp1, Bp9+Bp8, and Bp8 of the allotetraploids (**D**) *B. pinnatum* and (**E**) *B. phoenicoides* (2*n* = 28, *x* = 5 + 9). Only one homologue from a pair is shown. The colors of the BAC identifiers in the first column indicate the fluorochrome that was used (green, FITC; red, tetramethylrhodamine; yellow [false color], Alexa Fluor 647). The chromosomes were counterstained with DAPI (blue). The colored bars on the left and the BAC identifiers that were assigned to specific clones correspond to those on the cytogenetic maps in Figure 1 for the diploids *B. sylvaticum* and *B. pinnatum* and in Figure 3 for the allotetraploids *B. pinnatum* and *B. phoenicoides*. BACs Bd2S/2–6 and Bd2L/14–19 from Bd2 mapped to chromosomes Bp1, probes Bd2S/8 to Bd2L/13 on Bp8 and Bp9+Bp8. Probes Bd2S/6–8–10 and Bd2L/12–13–14 show the chromosomal breakpoints in the genome/subgenomes Bp compared to the chromosomal fusion points in the genome Bd. Probes Bd2S/10 + CEN + Bd2L/12 map to chromosomes Bp8 and Bp9+Bp8, whereas probes Bd2S/2 + CEN + Bd2L/19 hybridized to Bp1 thus indicating the presence of one NCF event in the Bd genome of *B. distachyon* that involved two ancestral chromosomes which were similar to Bp1 and Bp8 of the *x* = 9 genome/subgenome Bp.

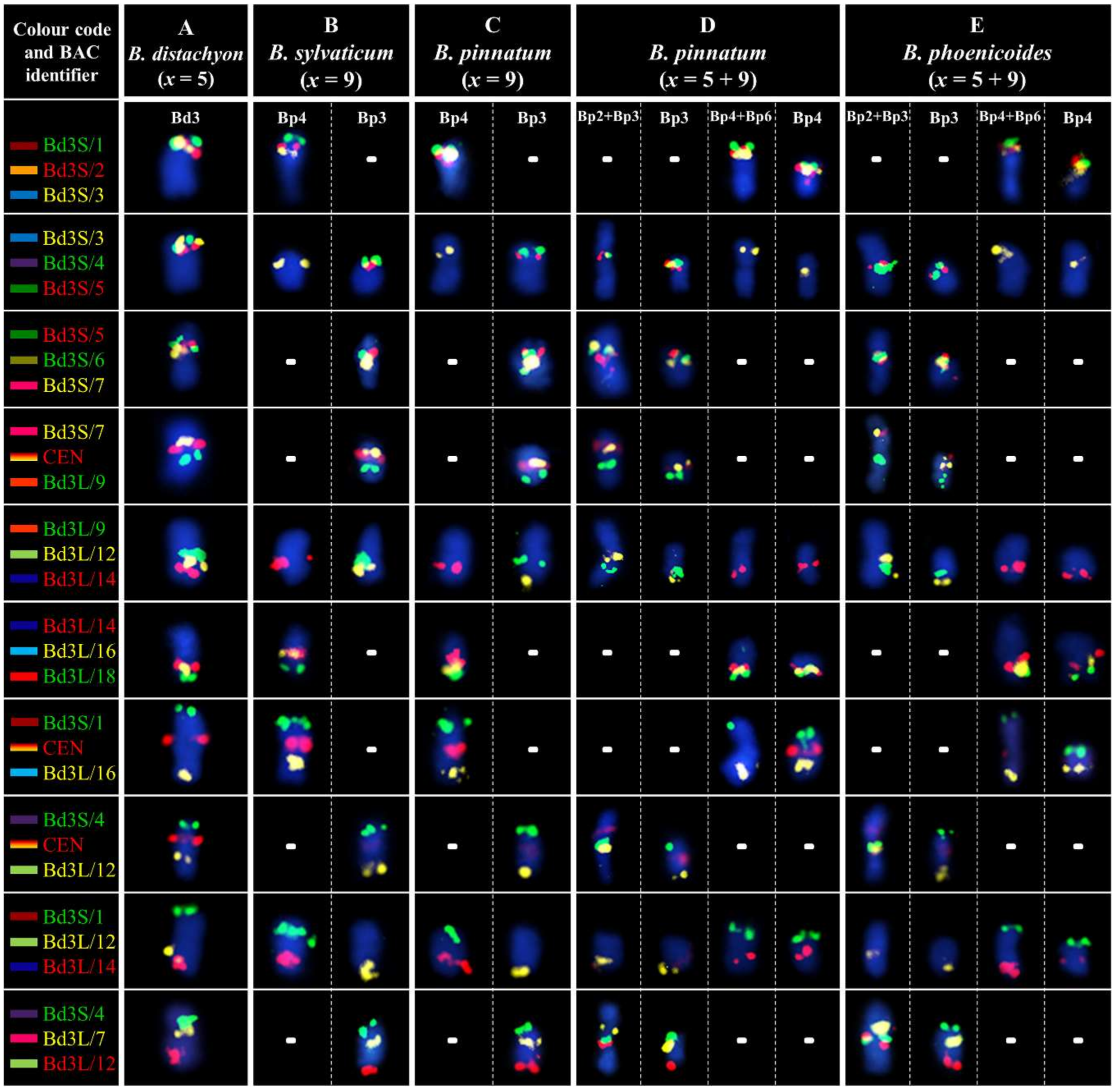

Figure S4. The BAC–FISH-based comparative chromosome barcoding with the clones derived from chromosome Bd3 of (**A**) *B. distachyon* ($2n$ = 10, x = 5) mapped to chromosomes Bp4 and Bp3 of the diploids (**B**) *Brachypodium sylvaticum* and (**C**) *B. pinnatum* (both $2n$ = 18, x = 9) and Bp2+Bp3, Bp3 chromosomes as well as Bp4+Bp6 and Bp4 chromosomes of the allotetraploids (**D**) *B. pinnatum* and (**E**) *B. phoenicoides* (both $2n$ = 28, x = 5 + 9). Only one homologue from a pair is shown. The colors of the BAC identifiers in the first column indicate the fluorochrome that was used (green, FITC; red, tetramethylrhodamine; yellow [false color], Alexa Fluor 647). The chromosomes were counterstained with DAPI (blue). The colored bars on the left and the BAC identifiers that were assigned to specific clones correspond to those on the cytogenetic maps in Figure 1 for the diploids *B. sylvaticum* and *B. pinnatum* and in Figure 3 for the allotetraploids *B. pinnatum* and *B. phoenicoides*. BACs Bd3S/1–3 and Bd3L/14–18 from Bd3 mapped to chromosomes Bp4+Bp6 and Bp4, probes Bd3S/4–7 and Bd3L/9–12 to Bp2+Bp3 and Bp3. Probes Bd3S/3–5, Bd3L/9–12–14 and Bd3S/1 + Bd3L/12–14 show the chromosomal breakpoints in the genome/subgenome Bp compared to the chromosomal fusion points in the genome Bd. Probes Bd3S/1 + CEN + Bd3L/16 map to chromosomes Bp4+Bp6 and Bp4, whereas probes Bd3S/7 + CEN + Bd3L/9, Bd3S/4 + CEN + Bd3L/12 and Bd3S/4–Bd3L/7–12 hybridized to chromosomes Bp2+Bp3 and Bp3, thus indicating the presence of one NCF event in the Bd genome of *B. distachyon* that involved two ancestral chromosomes that were similar to Bp4 and Bp3 of the x = 9 genome/subgenome Bp.

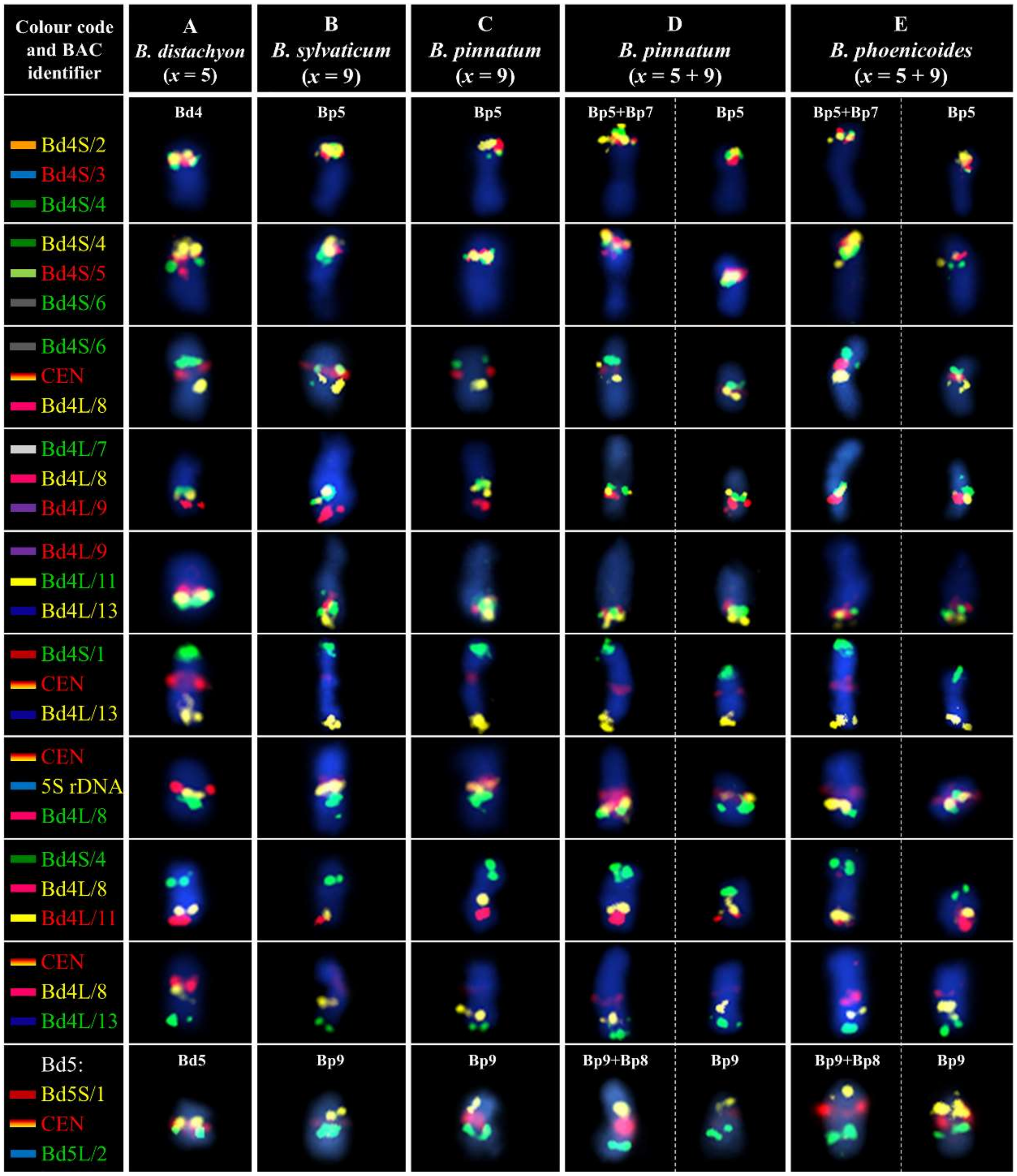

Figure S5. The BAC-FISH-based comparative chromosome barcoding (CCB) with the clones derived from chromosome Bd4 of (**A**) *Brachypodium distachyon* (2*n* = 10, *x* = 5) mapped to chromosomes Bp5 of the diploids (**B**) *B. sylvaticum* and (**C**) *B. pinnatum* (both 2*n* = 18, *x* = 9) and Bp5+Bp7 chromosome and Bp5 chromosome of the allotetraploids (**D**) *B. pinnatum* and (**E**) *B. phoenicoides* (both 2*n* = 28, *x* = 5 + 9). The lowest row shows CCB with the B5-derived probes of (**A**) the *B. distachyon* to chromosomes Bp9 of the diploids (**B**) *B. sylvaticum* and (**C**) *B. pinnatum* and Bp8+Bp9, Bp9 chromosomes of the allotetraploids (**D**) *B. pinnatum* and (**E**) *B. phoenicoides*. Only one homologue from a pair is shown. The colors of the BAC identifiers in the first column indicate the fluorochrome that was used (green, FITC; red, tetramethylrhodamine; yellow [false color], Alexa Fluor 647). The chromosomes were counterstained with DAPI (blue). The colored bars on the left and the BAC identifiers that were assigned to specific clones corresponded to those on the cytogenetic maps in Figure 1 for the diploids *B. sylvaticum* and *B. pinnatum* and in Figure 3 for the allotetraploids *B. pinnatum* and *B. phoenicoides*. All of the Bd4-derived BACs mapped in the same order along chromosome Bp5 thus indicating its high structural conservation in relation to Bd4. These clones also hybridized to the distal parts of both chromosome arms of the Bp5+Bp7 chromosome. The Bd5-derived BACs correspond to one ancestral chromosome and were mapped in a conserved order on chromosome Bp9 and in the distal parts of both chromosome arms of the Bp9+Bp8 chromosome.

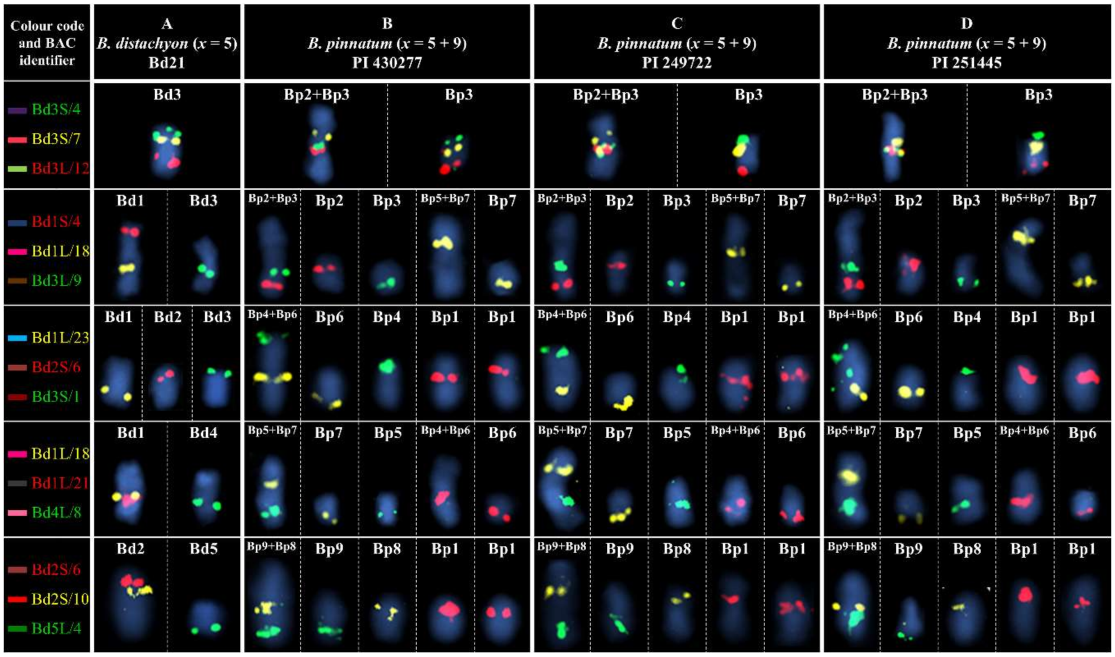

Figure S6. The BAC–FISH-based comparative chromosome barcoding with the clones derived from chromosomes Bd1–Bd5 of (**A**) *Brachypodium distachyon* ($2n$ = 10, x = 5) mapped to chromosomes in different genotypes of the allotetraploids *B. pinnatum* (**B**) PI 430277, (**C**) PI 249722, and (**D**) PI 251445 (all $2n$ = 28, x = 5 + 9). Only one homologue from a pair is shown. The colors of the BAC identifiers in the first column indicate the fluorochrome that was used (green, FITC; red, tetramethylrhodamine; yellow [false color], Alexa Fluor 647). The chromosomes were counterstained with DAPI (blue). The colored bars on the left and the BAC identifiers that were assigned to specific clones correspond to those on the cytogenetic maps in Figure 3. The specific distribution of Bd3S/4 + Bd3S/7 + Bd3L/12 BAC clones indicates the presence of a pericentric inversion in chromosome Bp2+Bp3. Probes Bd1S/4 (Bp2 in the x = 9 subgenome Bp) and Bd3L/9 (Bp3 in the x = 9 subgenome Bp) mapped together to the Bp2+Bp3 chromosome of the x = 5 subgenome Bp; this is evidence of one NCF that involved two ancestral chromosomes that were similar to Bp2 and Bp3. Probes Bd1L/23 (Bp4 in the x = 9 subgenome Bp) and Bd3S/1 (Bp6 in the x = 9 subgenome Bp) mapped together to the Bp4+Bp6 chromosome of the x = 5 subgenome Bp thus revealing one NCF event. Probes Bd1L/18 (Bp5 in the x = 9 subgenome Bp) and Bd4L/8 (Bp7 in the x = 9 subgenome Bp) hybridized together to the Bp5+Bp7 chromosome of the x = 5 subgenome Bp thus revealing another NCF that was specific to this subgenome. The same applies to probes Bd2S/10 (Bp8 in the x = 9 subgenome Bp) and Bd5L/4 (Bp9 in the x = 9 subgenome Bp) that mapped together to the Bp9+Bp8 chromosome of the x = 5 subgenome Bp. BAC clone Bd2S/6 mapped only to chromosome Bp1 of the x = 9 subgenome and Bp1 of the x = 5 subgenome thus indicating a lack of the chromosomal fusions that are involved in the formation of these chromosomes. No intraspecific variation was revealed among the studied genotypes.

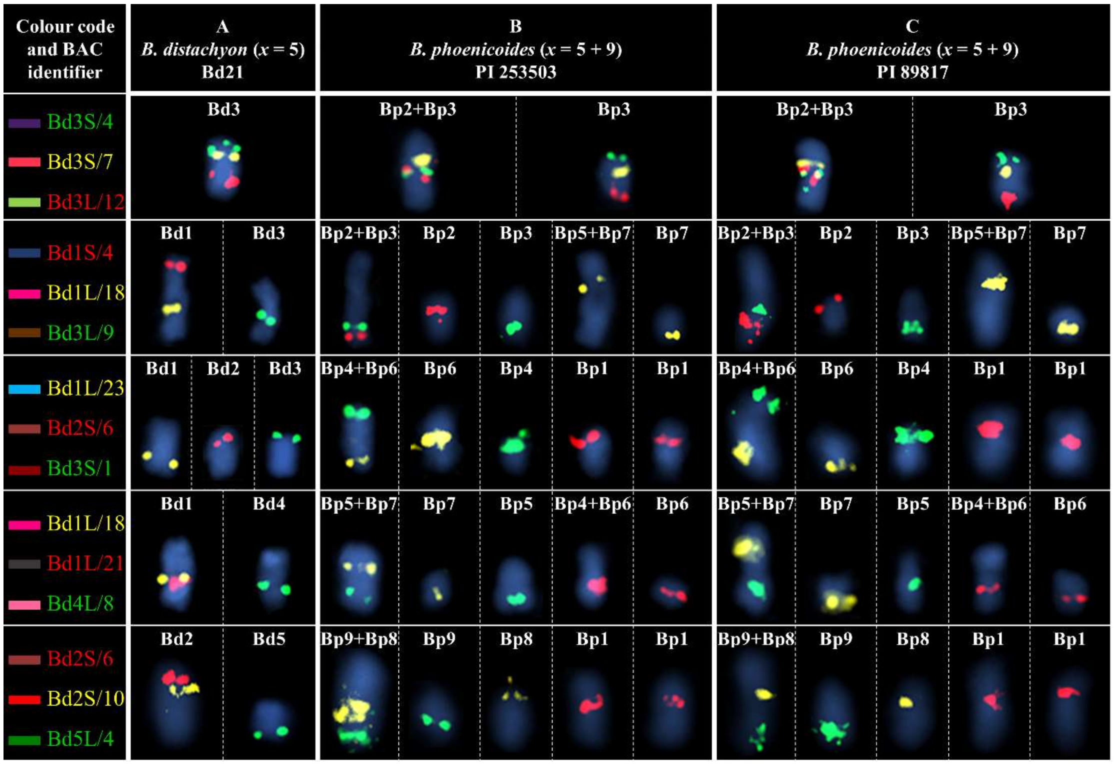

Figure S7. The BAC–FISH-based comparative chromosome barcoding with the clones derived from chromosomes Bd1–Bd5 of (**A**) *Brachypodium distachyon* ($2n = 10$, $x = 5$) mapped to the chromosomes in the different genotypes of the allotetraploids *B. phoenicoides* (**B**) PI 253503 and (**C**) PI 89817 (all $2n = 28$, $x = 5 + 9$). Only one homologue from a pair is shown in each cell. The colors of the BAC identifiers in the first column indicate the fluorochrome that was used (green, FITC; red, tetramethylrhodamine; yellow [false color], Alexa Fluor 647). The chromosomes were counterstained with DAPI (blue). The colored bars on the left and the BAC identifiers that were assigned to specific clones correspond to those on the cytogenetic maps in Figure 3. The specific distribution of Bd3S/4 + Bd3S/7 + Bd3L/12 BAC clones indicates the presence of a pericentric inversion in chromosome Bp2+Bp3. Probes Bd1S/4 (Bp2 in the $x = 9$ subgenome Bp) and Bd3L/9 (Bp3 in the $x = 9$ subgenome Bp) mapped together to the Bp2+Bp3 chromosome of the $x = 5$ subgenome Bp thus providing evidence for one NCF that involved two ancestral chromosomes that were similar to Bp2 and Bp3. Probes Bd1L/23 (Bp4 in the $x = 9$ subgenome Bp) and Bd3S/1 (Bp6 in the $x = 9$ subgenome Bp) mapped together to the Bp4+Bp6 chromosome of the $x = 5$ subgenome Bp thus revealing one NCF event. Probes Bd1L/18 (Bp5 in the $x = 9$ subgenome Bp) and Bd4L/8 (Bp7 in the $x = 9$ subgenome Bp) hybridized together to the Bp5+Bp7 chromosome of the $x = 5$ subgenome Bp thus revealing another NCF specific for this subgenome. The same applies to probes Bd2S/10 (Bp8 in the $x = 9$ subgenome Bp) and Bd5L/4 (Bp9 in the $x = 9$ subgenome Bp) that mapped together to the Bp9+Bp8 chromosome of the $x = 5$ subgenome Bp. BAC clone Bd2S/6 mapped only to chromosome Bp1 of the $x = 9$ subgenome and Bp1 of the $x = 5$ subgenome thus indicating a lack of the chromosomal fusions that are involved in the formation of these chromosomes. No intraspecific variation was revealed among the studied genotypes.

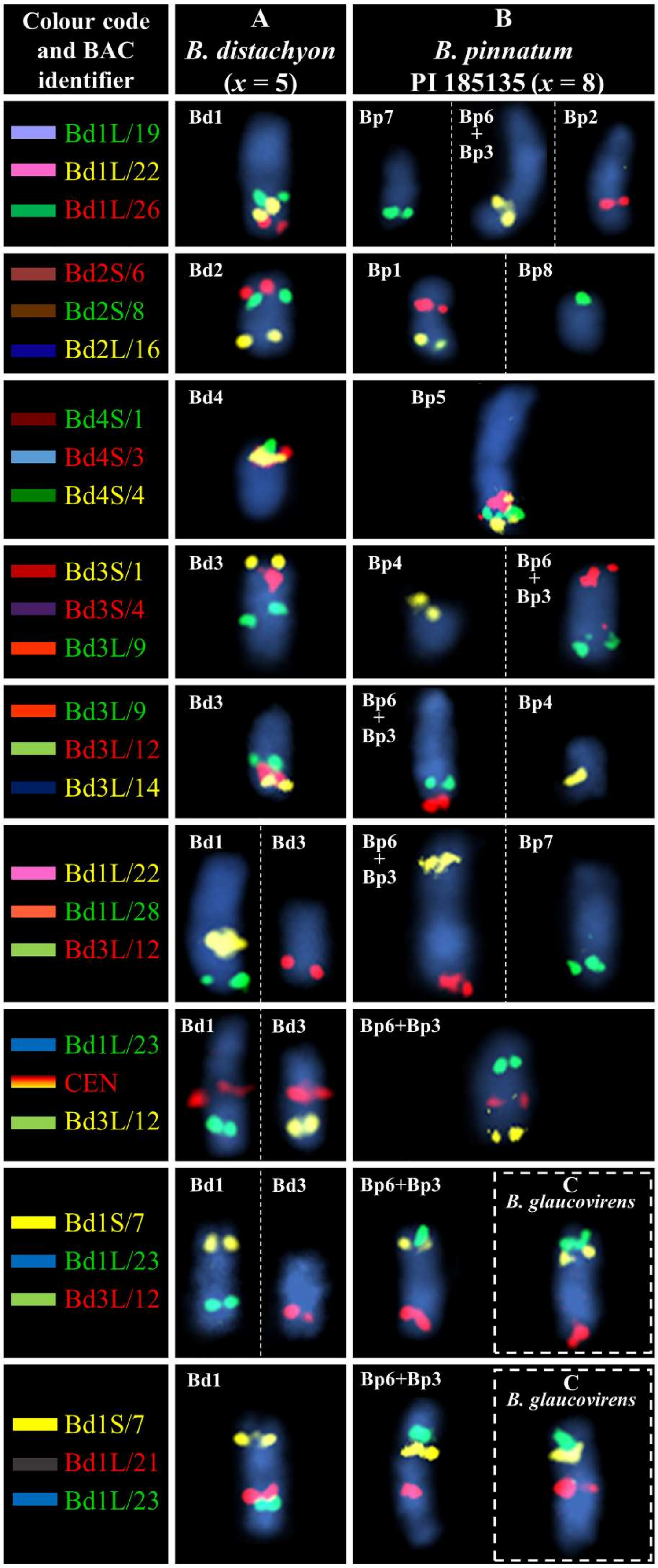
Colour code and BAC identifier
A
B. distachyon (x = 5)
B
B. pinnatum PI 185135 (x = 8)
Bd1L/19
Bd1L/22
Bd1L/26
Bd1
Bp7
Bp6 + Bp3
Bp2
Bd2S/6
Bd2S/8
Bd2L/16
Bd2
Bp1
Bp8
Bd4S/1
Bd4S/3
Bd4S/4
Bd4
Bp5
Bd3S/1
Bd3S/4
Bd3L/9
Bd3
Bp4
Bp6 + Bp3
Bd3L/9
Bd3L/12
Bd3L/14
Bd3
Bp6 + Bp3
Bp4
Bd1L/22
Bd1L/28
Bd3L/12
Bd1
Bd3
Bp6 + Bp3
Bp7
Bd1L/23
CEN
Bd3L/12
Bd1
Bd3
Bp6+Bp3
Bd1S/7
Bd1L/23
Bd3L/12
Bd1
Bd3
Bp6+Bp3
C
B. glaucovirens
Bd1S/7
Bd1L/21
Bd1L/23
Bd1
Bp6+Bp3
C
B. glaucovirens

Figure S8. The BAC–FISH-based comparative chromosome barcoding with the clones derived from chromosomes Bd1–Bd4 of (**A**) *Brachypodium distachyon* (2*n* = 10, *x* = 5) mapped to the chromosomes of the diploids (**B**) *B. pinnatum* PI 185135 and (**C**) *B. glaucovirens* (both 2*n* = 16, *x* = 8). Only one homologue from a pair is shown in each cell. The colors of the BAC identifiers in the first column indicate the fluorochrome that was used (green, FITC; red, tetramethylrhodamine; yellow [false color], Alexa Fluor 647). The chromosomes were counterstained with DAPI (blue). The colored bars on the left and the BAC identifiers that were assigned to specific clones correspond to those on the cytogenetic maps in Figure 2. Probes Bd1L/22 (Bp6 in the *x* = 9 subgenome Bp) and Bd3L/12 (Bp3 in the *x* = 9 subgenome Bp) mapped together to the Bp6+Bp3 chromosome of the *x* = 5 subgenome Bp. The same applied to probes Bd1L/23 (Bp6 in the *x* = 9 subgenome Bp) and Bd3L/12 (Bp3 in the *x* = 9 subgenome Bp). The specific distribution of BACs Bd1S/7 + Bd1L/23 + Bd3L/12 revealed the presence of an end-to-end fusion in the 2*n* = 16 diploid perennials (**B**,**C**) that involved two ancestral chromosomes that were similar to Bp6 and Bp3 of the *x* = 9 subgenome Bp, which led to the formation of the specific Bp6+Bp3 chromosome. The chromosomal arrangement of Bd1S/7 + Bd1L/21 + Bd1L/23 indicated the presence of a pericentric inversion within the Bp6+Bp3 chromosome.

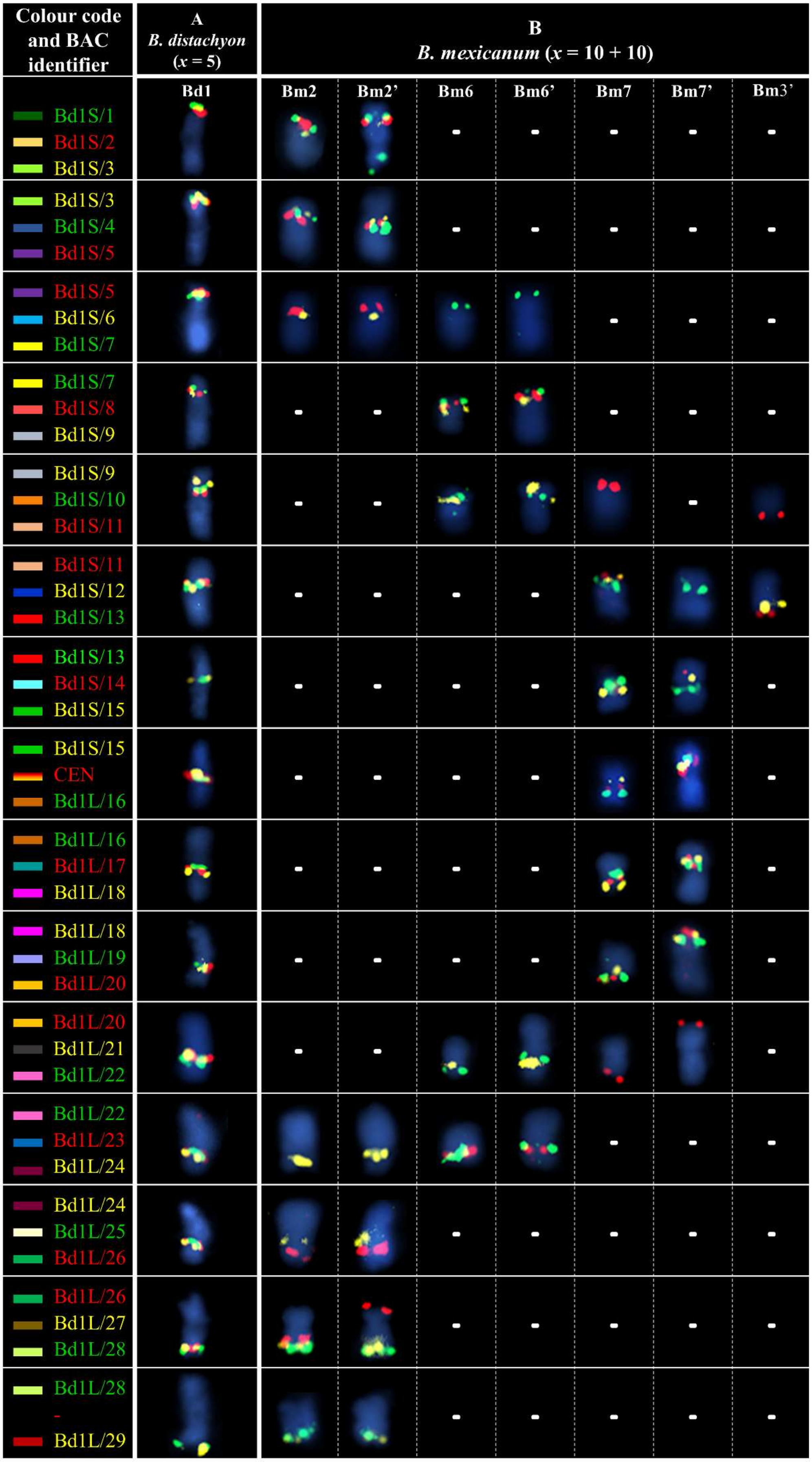
Colour code and BAC identifier
A
B. distachyon (x = 5)
B
B. mexicanum (x = 10 + 10)
Bd1
Bm2
Bm2'
Bm6
Bm6'
Bm7
Bm7'
Bm3'
Bd1S/1
Bd1S/2
Bd1S/3
Bd1S/3
Bd1S/4
Bd1S/5
Bd1S/5
Bd1S/6
Bd1S/7
Bd1S/7
Bd1S/8
Bd1S/9
Bd1S/9
Bd1S/10
Bd1S/11
Bd1S/11
Bd1S/12
Bd1S/13
Bd1S/13
Bd1S/14
Bd1S/15
Bd1S/15
CEN
Bd1L/16
Bd1L/16
Bd1L/17
Bd1L/18
Bd1L/18
Bd1L/19
Bd1L/20
Bd1L/20
Bd1L/21
Bd1L/22
Bd1L/22
Bd1L/23
Bd1L/24
Bd1L/24
Bd1L/25
Bd1L/26
Bd1L/26
Bd1L/27
Bd1L/28
Bd1L/28
-
Bd1L/29

Figure S9. The BAC-FISH-based comparative chromosome barcoding with the clones derived from chromosome Bd1 of (**A**) *Brachypodium distachyon* (2*n* = 10, *x* = 5) mapped to chromosomes Bm2, Bm2′, Bm6, Bm6′, Bm7, Bm7′, and Bm3′ of (**B**) *B. mexicanum* (2*n* = 40, *x* = 10 + 10). Only one homologue from a pair is shown. The colors of the BAC identifiers in the first column indicate the fluorochrome that was used (green, FITC; red, tetramethylrhodamine; yellow [false color], Alexa Fluor 647). The chromosomes were counterstained with DAPI (blue). The colored bars on the left and the BAC identifiers that were assigned to specific clones correspond to those on the cytogenetic maps in Figure 4. BACs Bd1S/1–6 and Bd1L/24–29 mapped to chromosomes Bm2 and Bm2′, probes Bd1S/7–10 and Bd1L/21–23 to Bm6 and Bm6′. Probes Bd1S/11–Bd1L/20 mapped to Bm7, whereas probes Bd1S/13–Bd1L/20 mapped to Bm7′, and probes Bd1S/11–12 mapped to Bm3′. BAC clones Bd1S/5–7, Bd1S/9–11, and Bd1S/11–13 as well as Bd1L/20–22 and Bd1L/22–24 show chromosomal breakpoints in the subgenomes Bm and Bm′ compared to the chromosomal fusion points in the genome Bd. BAC clone Bd1S/1 had a second hybridization signal on the opposite arms of Bm2′. Within the BAC triplet Bd1L/26–28, clone Bd1L/26 mapped to the opposite arm of Bm2′ compared to Bm2. Probe triplets Bd1L/20 + Bd1L/21 + Bd1L/22 were characterized by an inverted arrangement of clones Bd1L/21–23 thus indicating the presence of a paracentric inversion in the long arm of Bm6′ compared to Bm6. Comparative mapping of the Bd1-derived clones to *B. mexicanum* chromosomes is continued in Figure S10.

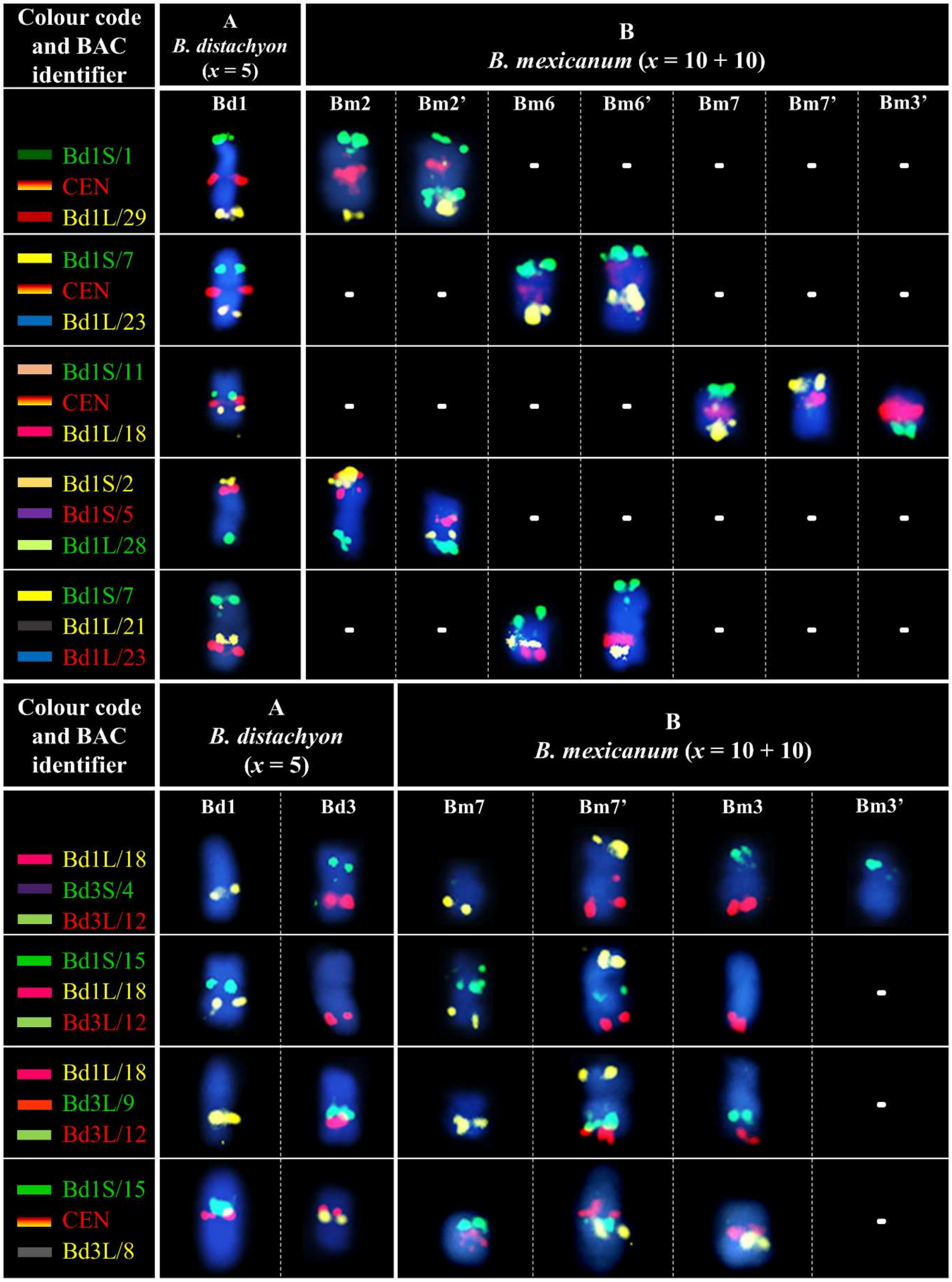

Figure S10. The BAC-FISH-based comparative chromosome barcoding with the clones derived from chromosomes Bd1 and Bd3 of (**A**) *Brachypodium distachyon* (2*n* = 10, *x* = 5) mapped to chromosomes Bm2, Bm2′, Bm6, Bm6′, Bm7, Bm7′, Bm3 and Bm3′ of (**B**) *B. mexicanum* (2*n* = 40, *x* = 10 + 10). Only one homologue from a pair is shown. The colors of the BAC identifiers in the first column indicate the fluorochrome that was used (green, FITC; red, tetramethylrhodamine; yellow [false color], Alexa Fluor 647). The chromosomes were counterstained with DAPI (blue). The colored bars on the left and the BAC identifiers that were assigned to specific clones correspond to those on the cytogenetic maps in Figure 4. BACs Bd1S/1 + CEN + Bd1L/29 mapped to chromosomes Bm2 and Bm2′, probes Bd1S/7 +

CEN + Bd1L/23 hybridized to chromosomes Bm6 and Bm6′, whereas clones Bd1S/11 + CEN + Bd1L/18 mapped together only on chromosome Bm7 thus indicating the presence of two NCF events in the Bd genome of *B. distachyon* that involved three ancestral chromosomes that were similar to Bm2, Bm6, and Bm7 of the $x = 10$ subgenome Bm. The arrangement of the probe triplets Bd1S/1 + CEN + Bd1L/29 and Bd1S/2 + Bd1S/5 + Bd1L/28 indicated a large pericentric inversion combined with a duplication of the region hybridizing with clone Bd1S/1 on chromosome Bm2′. The probe triplets Bd1S/7 + Bd1L/21 + Bd1L/23 were characterized by an inverted arrangement of clones Bd1L/21 and Bd1L/23 clones thus indicating the presence of a paracentric inversion in the long arm of Bm6′ compared to Bm6. BACs Bd1S/11 and Bd1L/18 hybridized together to chromosome Bm7 and separately on Bm7′ and Bm3′. Probe triplets Bd1L/18 + Bd3S/4 + Bd3L/12 and Bd1S/15 + Bd1L/18 + Bd3L/12 as well as Bd1L/18 + Bd3L/9 + Bd3L/12 and Bd1S/15 + CEN + Bd3L/8 derived from chromosomes Bd1 and Bd3 hybridized together to chromosome Bm7′, revealing the occurrence a reciprocal translocation between this chromosome and Bm3′.

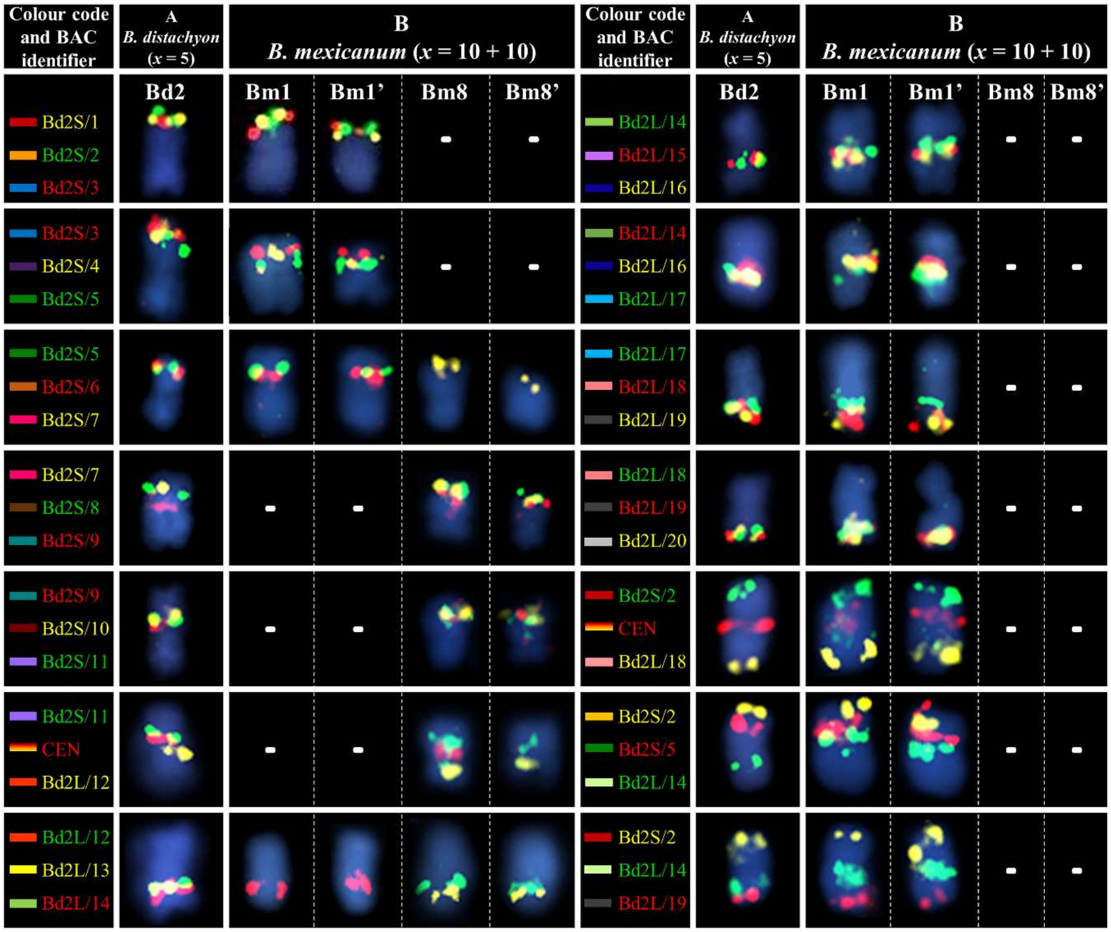

Figure S11. The BAC–FISH-based comparative chromosome barcoding with the clones derived from chromosome Bd2 of (**A**) *Brachypodium distachyon* (2*n* = 10, *x* = 5) mapped to chromosomes Bm1, Bm1′, Bm8, and Bm8′ of (**B**) *B. mexicanum* (2*n* = 40, *x* = 10 + 10). Only one homologue from a pair is shown. The colors of the BAC identifiers in the first column indicate the fluorochrome that was used (green, FITC; red, tetramethylrhodamine; yellow [false color], Alexa Fluor 647). The chromosomes were counterstained with DAPI (blue). The colored bars on the left and the BAC identifiers that were assigned to specific clones correspond to those on the cytogenetic maps in Figure 4. BACs Bd2S/1–6 and Bd2L/14–20 mapped to chromosomes Bm1 and Bm1′, while the probes from Bd2S/7 to Bd2L/13 to Bm8 and Bm8′. The BAC triplets Bd2S/5–6–7 and Bd2L/12–13–14 had chromosomal breakpoints in the subgenomes Bm and Bm′ compared to the chromosomal fusion points in the genome Bd. Probes Bd2S/11 + CEN + Bd2L/12 hybridized to chromosomes Bm8 and Bm8′, whereas the BAC triplets Bd2S/2 + CEN + Bd2L/18, Bd2S/2 + Bd2S/5 + Bd2L/14, and Bd2S/2 + Bd2L/14 + Bd2L/19 hybridized to chromosomes Bm1 and Bm1′ thus indicating the presence of one NCF event in the Bd genome of *B. distachyon* that involved two ancestral chromosomes that were similar to Bm1 and Bm8 or to Bm1′ and Bm8′ of subgenomes Bm.

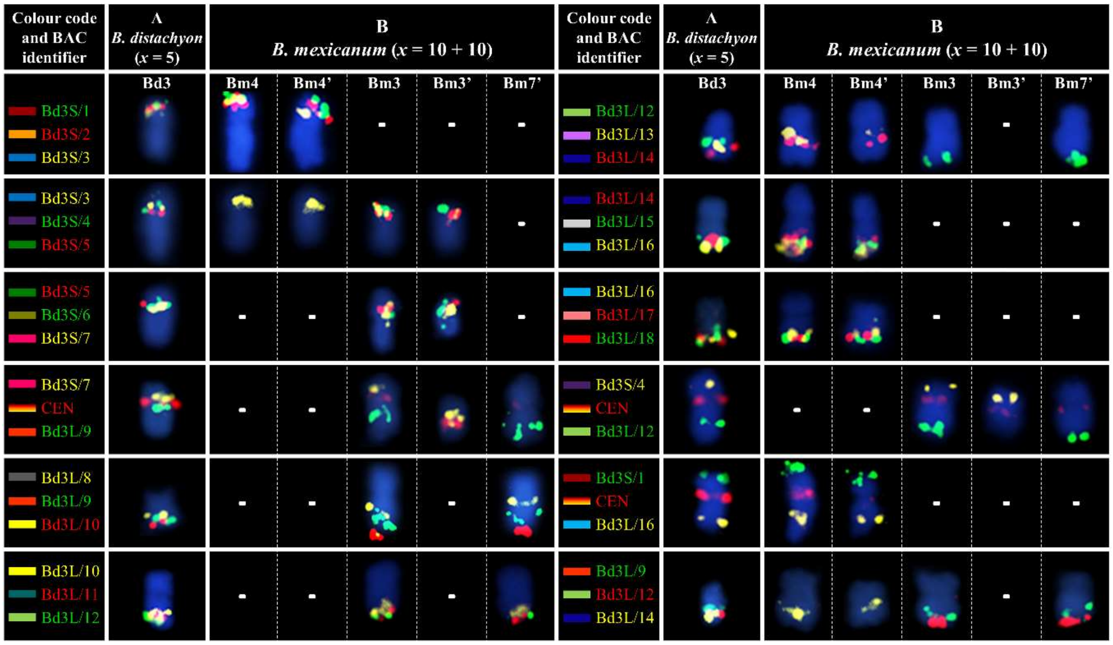

Figure S12. The BAC–FISH-based comparative chromosome barcoding with the clones derived from chromosome Bd3 of (**A**) *Brachypodium distachyon* (2*n* = 10, *x* = 5) mapped to chromosomes Bm4, Bm4′, Bm3, Bm3′ and Bm7′ of (**B**) *B. mexicanum* (2*n* = 40, *x* = 10 + 10). Only one homologue from a pair is shown. The colors of the BAC identifiers in the first column indicate the fluorochrome that was used (green, FITC; red, tetramethylrhodamine; yellow [false color], Alexa Fluor 647). The chromosomes were counterstained with DAPI (blue). The colored bars on the left and the BAC identifiers that were assigned to specific clones correspond to those on the cytogenetic maps in Figure 4. BACs Bd3S/1–3 and Bd3L/13–18 mapped to chromosomes Bm4 and Bm4′. Probes Bd3S/4–7 and Bd3L/8–12 mapped together to chromosome Bm3. Additionally, clones Bd3S/4–7 hybridized also to Bm3′, whereas probes Bd3L/8–12 hybridized to chromosome Bm7′, indicating the presence of a reciprocal translocation between chromosomes Bm7′ and Bm3′. Probe triplets Bd3S/3–5, Bd3S/7 + CEN + Bd3L/9, Bd3L/12–14, Bd3S/4 + CEN + Bd3L/12, and Bd3L/9–12–14 show the chromosomal breakpoints in the subgenomes Bm and Bm′ compared to the chromosomal fusion points in the genome Bd. Probes Bd3S/1 + CEN + Bd3L/16 hybridized to chromosomes Bm4 and Bm4′, whereas probes Bd3S/7 + CEN + Bd3L/9 and Bd3S/4 and Bd3L/12 hybridized to chromosome Bm3 thus indicating the presence of one NCF event in the Bd genome of *B. distachyon* that involved two ancestral chromosomes similar to Bm4 or Bm4′ and Bm3 of subgenomes Bm.

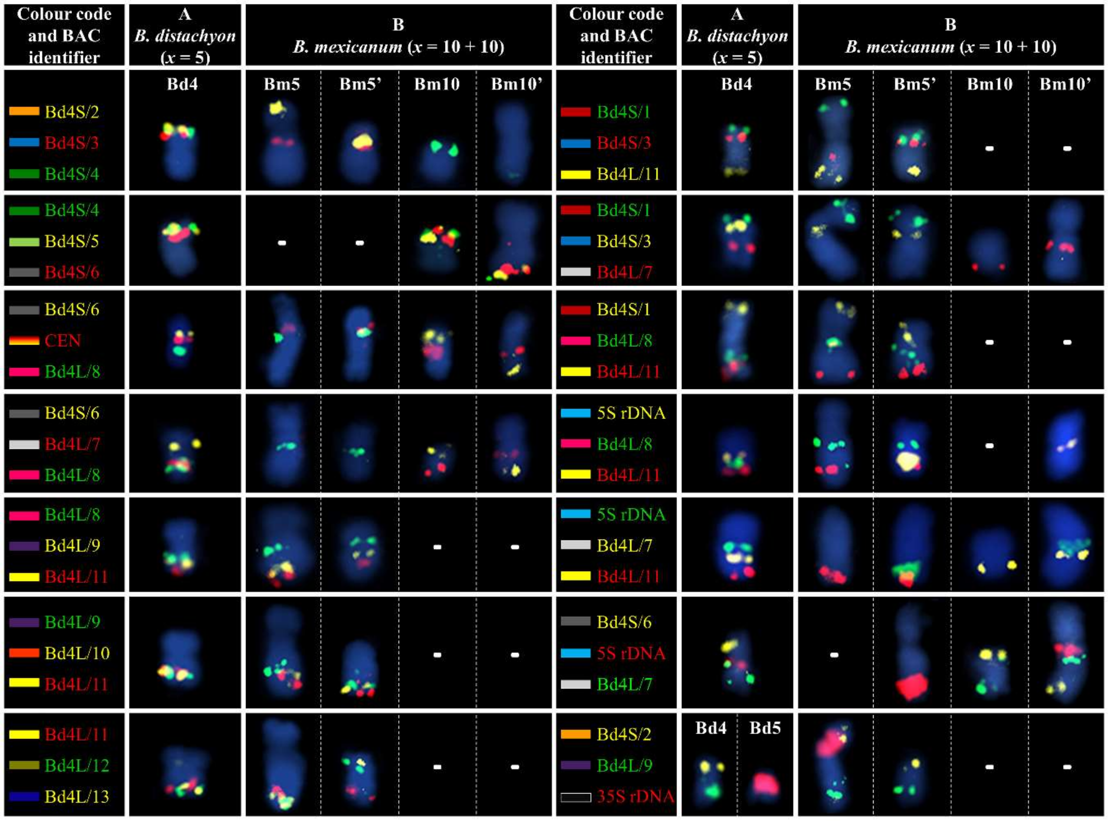

Figure S13. The BAC–FISH-based comparative chromosome barcoding with the clones derived from chromosome Bd4 of (**A**) *Brachypodium distachyon* (2*n* = 10, *x* = 5) mapped to chromosomes Bm5, Bm5′, Bm10, and Bm10′ of (**B**) *B. mexicanum* (2*n* = 40, *x* = 10 + 10). Only one homologue from a pair is shown. The colors of the BAC identifiers in the first column indicate the fluorochrome that was used (green, FITC; red, tetramethylrhodamine; yellow [false color], Alexa Fluor 647). The chromosomes were counterstained with DAPI (blue). The colored bars on the left and the BAC identifiers that were assigned to specific clones correspond to those on the cytogenetic maps in Figure 4. BACs Bd4S/1–3 and Bd4L/8–13 mapped to chromosome Bm5 and Bm5′, while probes Bd4S/4–6 and Bd4L/7 to Bm10 and Bm10′. Bm5 was the only 35S rDNA-bearing chromosome in *B. mexicanum* that had a distinct secondary constriction on its short arm. Chromosomes Bm5′ and Bm10′ carried 5S rDNA loci on their long arms; these loci differed in terms of their localization and size. In contrast to the reference chromosome Bd4, Bm10 did not have a 5S rDNA locus. The distribution of clones Bd4L/12–13 on the short arm of chromosome Bm5′ in relation to probe Bd4L/11 mapped to the opposite chromosome arm, which indicates the presence of an intrachromosomal translocation that is connected with a paracentric inversion. BAC clone Bd4S/4 hybridized to chromosomes Bm10 and Bm10′, giving a strong signal on the former and a very weak signal on the latter. The different order of Bd4S/6 + 5S rDNA + Bd4L/7 loci in Bm10′ compared to Bd4 suggests the occurrence of a small pericentric inversion that involved the proximal region of Bm10′. Probes Bd4S/2–4, Bd4S/6 + CEN + Bd4L/8, and Bd4S/6 + Bd4L/7–8 and Bd4S/1 + Bd4S/3 + Bd4L/7 had chromosomal breakpoints in the subgenomes Bm and Bm′ compared to the chromosomal fusion points in the genome Bd. Probes Bd4S/1 + Bd4L/8 + Bd4L/11 and Bd4S/1 + Bd4S/3 + Bd4L/11 mapped to chromosome Bm5 and Bm5′, whereas the probes from Bd4S/4–6 to Bd4L/7 hybridized to chromosomes Bm10 and Bm10′ thus indicating the presence of one NCF event in the Bd genome of *B. distachyon* that involved two ancestral chromosomes that were similar to Bm5 and Bm10 or Bm5′ and Bm10′ of subgenomes Bm and Bm′.

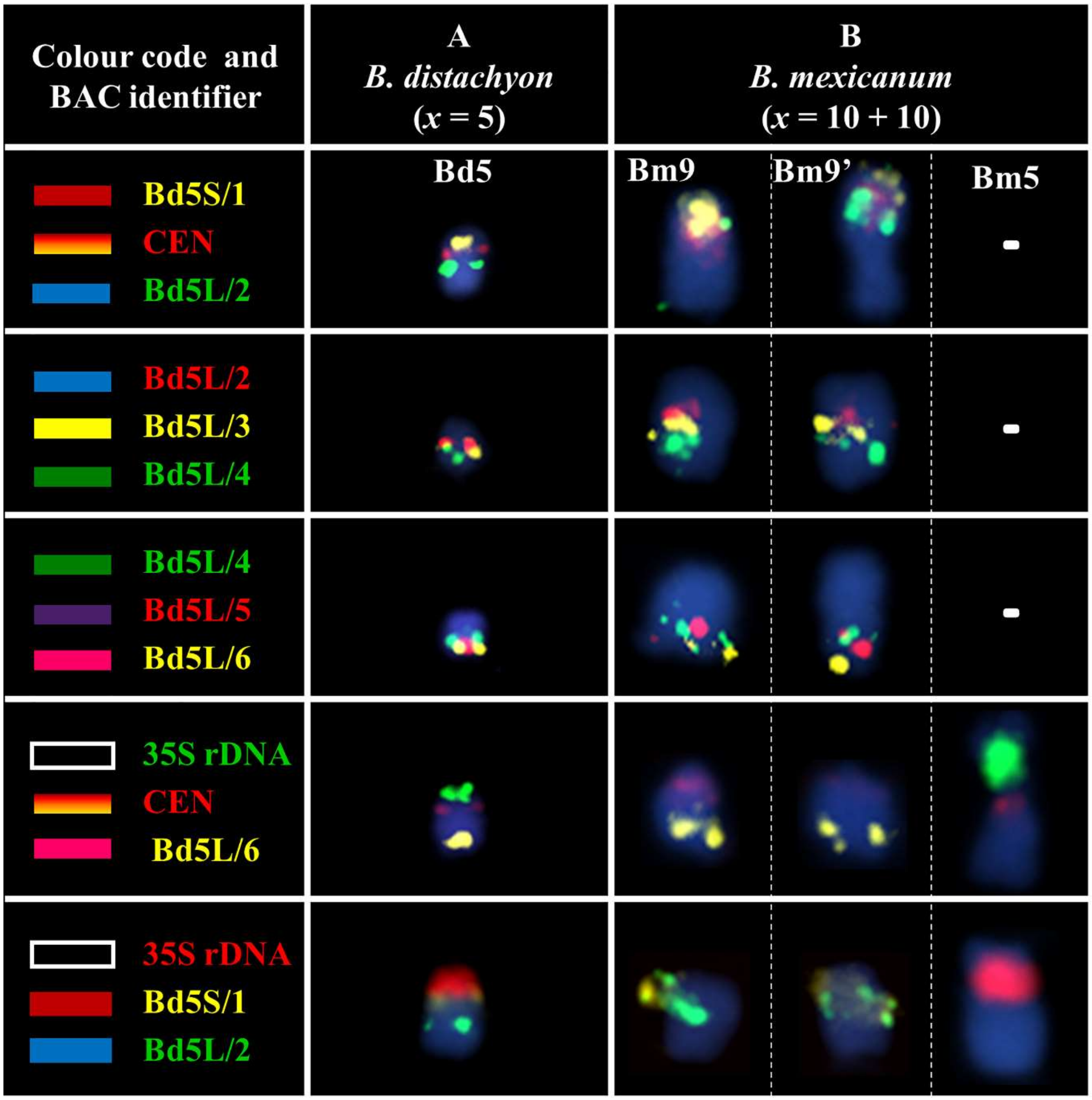

Figure S14. The BAC–FISH-based comparative chromosome barcoding with the clones derived from chromosome Bd5 of (**A**) *Brachypodium distachyon* (2*n* = 10, *x* = 5) mapped to chromosomes Bm9 and Bm9′ of (**B**) *B. mexicanum* (2*n* = 40, *x* = 10 + 10). Only one homologue from a pair is shown. The colors of the BAC identifiers in the first column indicate the fluorochrome that was used (green, FITC; red, tetramethylrhodamine; yellow [false color], Alexa Fluor 647). The chromosomes were counterstained with DAPI (blue). The colored bars on the left and the BAC identifiers that were assigned to specific clones correspond to those on the cytogenetic maps in Figure 4.
